# Tumor microenvironmental influences on dendritic cell and T cell function: A focus on clinically relevant immunologic and metabolic checkpoints

**DOI:** 10.1002/ctm2.37

**Published:** 2020-05-08

**Authors:** Kristian M. Hargadon

**Affiliations:** ^1^ Hargadon Laboratory Department of Biology Hampden‐Sydney College Hampden‐Sydney Virginia USA

**Keywords:** adoptive cell transfer, cancer, checkpoint blockade, dendritic cell, immune suppression, metabolism, T cell, tumor microenvironment, vaccine

## Abstract

Cancer immunotherapy is fast becoming one of the most promising means of treating malignant disease. Cancer vaccines, adoptive cell transfer therapies, and immune checkpoint blockade have all shown varying levels of success in the clinical management of several cancer types in recent years. However, despite the clinical benefits often achieved by these regimens, an ongoing problem for many patients is the inherent or acquired resistance of their cancer to immunotherapy. It is now appreciated that dendritic cells and T lymphocytes both play key roles in antitumor immune responses and that the tumor microenvironment presents a number of barriers to the function of these cells that can ultimately limit the success of immunotherapy. In particular, the engagement of several immunologic and metabolic checkpoints within the hostile tumor microenvironment can severely compromise the antitumor functions of these important immune populations. This review highlights work from both preclinical and clinical studies that has shaped our understanding of the tumor microenvironment and its influence on dendritic cell and T cell function. It focuses on clinically relevant targeted and immunotherapeutic strategies that have emerged from these studies in an effort to prevent or overcome immune subversion within the tumor microenvironment. Emphasis is also placed on the potential of next‐generation combinatorial regimens that target metabolic and immunologic impediments to dendritic cell and T lymphocyte function as strategies to improve antitumor immune reactivity and the clinical outcome of cancer immunotherapy going forward.

## 1 BACKGROUND

Over the past decade, the emergence of immunotherapy as a viable and promising treatment option for both solid and blood‐based malignancies has revolutionized the therapeutic landscape of cancer, resulting in unprecedented achievements in the clinical management of this disease. During this short period, significant improvements over traditional forms of cancer therapy have been realized in terms of both the rate and duration of clinical responses, and immunotherapeutic regimens have now become standard‐of‐care treatment for many cancer types.^1^, ^2^ However, as has often been the case with traditional approaches to cancer treatment, many patients who initially respond to immune‐based therapies eventually experience disease relapse, and still there are others who fail to respond to immunotherapy at all. In light of these disparate clinical outcomes, significant efforts have been made to better understand factors that influence the quality of antitumor immune responses and the clinical efficacy of regimens that rely on their induction and maintenance. In this regard, several forms of innate and adaptive immune resistance have now been described and are linked to tumor cell‐intrinsic and extrinsic factors that ultimately interfere with the complex cross‐talk between cancer cells and the diverse immune/non‐immune cell populations that participate in and regulate antitumor immune responses. Central to many of these resistance mechanisms is the role played by the hostile tumor microenvironment (TME) in preventing or dampening antitumor immunity. In particular, dendritic cells (DC) and T lymphocytes, two immune cell populations critical to the efficacy of antitumor immune responses, are subject to regulation by various immunologic and metabolic checkpoints within the TME. This review highlights our current understanding of these checkpoints and the limitations they place on the immune functions of DC and T cells, and it describes combinatorial strategies for overcoming these barriers to immune function that have the potential to enhance the quality and longevity of anticancer immune responses and to improve the clinical outcome of cancer immunotherapy going forward.

## 2 DC/T CELL COLLABORATION IN ANTITUMOR IMMUNITY

The contribution of T lymphocytes to immune protection from cancer has long been appreciated. At the turn of the century, seminal studies in murine models first revealed the importance of these cells and effector molecules derived from them in the immunologic control of various tumors.^3^, ^4^ Prior to and during this time, evidence supporting T cell reactivity against human cancers also emerged from clinical observations of spontaneous tumor regression in patients experiencing T cell‐driven autoimmune disease in the same tissue from which their cancer was derived (ie, vitiligo in melanoma patients) as well as in some patients exhibiting antigen (Ag)‐specific T cell infiltration of their tumors.^5^, ^6^ While the role of DC in regulating T cell tolerance and immunity to pathogens has also long been appreciated,^7^ the ways in which these cells influence antitumor immune responses have only more recently become apparent. Following significant efforts to better understand DC function in the context of cancer, these cells are now known to contribute to various stages of the antitumor T cell response, from induction of an Ag‐specific response in secondary lymphoid organs to recruitment of effector cells into tumor tissue to maintenance/restimulation of cytotoxic T lymphocytes (CTL) within the TME.^8^

In most cases, successful priming of antitumor T cell responses requires migration of tumor‐infiltrating DC (TIDC) to regional lymph nodes, where they are able to cross‐present Ag from phagocytosed tumor cells to naïve CD8^+^ T lymphocytes. Type 1 conventional DC (cDC1), marked by expression of CD103 in mice and CD141 in humans, are particularly relevant to this cross‐priming,^9^, ^10^ which depends not only on DC access to tumor tissue for Ag acquisition but also appropriate maturation and activation of these cells into potent immunostimulatory Ag‐presenting cells (APC). In addition to priming CD8^+^ T cell responses within tumor‐draining lymph nodes, the cDC1 subset of DC also regulates effector CTL trafficking to tumors, as those DC that remain within tumor tissue can secrete CXCL9/CXCL10 chemokines to attract CXCR3^+^ effector CTL,^11^, ^12^ a process relevant to both endogenous antitumor T cell responses as well as therapeutic regimens that rely on administration of exogenously activated CTL. Finally, cDC1 as well as other DC subsets have the potential to produce high levels of IL‐12 and type I IFN,^13^, ^14^, ^15^ immunostimulatory cytokines that not only promote DC‐mediated cross‐priming of antitumor T cell responses but that also likely help to maintain CTL effector function within the TME when produced by intratumoral DC. While this complex interplay between DC and T lymphocytes over the course of an antitumor immune response therefore enables the initiation, direction, and maintenance of a T cell response to cancer, it also provides multiple opportunities for tumors to circumvent immune‐mediated destruction. Indeed, several immunologic and metabolic checkpoints that restrict the functions of DC and T lymphocytes within the TME have recently been discovered and found to limit the efficacy of antitumor immune responses. Despite the significance of these barriers to successful antitumor immune reactivity, though, advances in our understanding of these immune‐disrupting pathways and the mechanisms underlying their immunoregulatory functions are now paving the way for therapeutic strategies that aim to: (a) restore proper communication between DC and T lymphocytes in the context of cancer and (b) promote the robust antitumor activities capable of being mediated by these cells.

## 3 INNATE IMMUNE CHECKPOINTS INFLUENCING DC FUNCTION IN THE TUMOR MICROENVIRONMENT

### 3.1 CD47 and SIRPα

CD47 is a cell surface glycoprotein expressed on nearly all cell types that serves as a marker of self to the innate immune system. Though first recognized for its role in inhibiting host cell phagocytosis by macrophages,^16^ CD47 has since emerged as a prominent “don't eat me” signal to various phagocytic cell populations that express the signal regulatory protein‐α (SIRPα) receptor.^17^, ^18^, ^19^ Importantly, CD47 expression is upregulated on tumor cells of several cancer types^20^ and has been shown to promote evasion of phagocytosis by both macrophages and DC.^21^, ^22^ Based on these findings, CD47 has become an attractive target for cancer therapy, and several studies have now confirmed the antitumor efficacy of its blockade,^23^, ^24^, ^25^, ^26^ which not only promotes tumor cell clearance by macrophages and DC but also supports therapeutic regimens linked to adaptive antitumor immunity.^27^ Indeed, the therapeutic efficacy of CD47 blockade has been shown to be T cell‐dependent,^28^ and DC in particular are critical to the enhanced antitumor T cell response resulting from this therapy. Whereas both macrophages and DC benefit from CD47 blockade in terms of phagocytic uptake of tumor cells, Xu *et al* recently found that CD47 blockade also selectively enhances innate immune sensing of tumor mitochondrial DNA (mtDNA) in DC by activating NOX2 and limiting the phagosomal acidification that otherwise degrades this DNA within macrophages. The increased stability of phagocytosed tumor mtDNA within DC following CD47 blockade enables its subsequent release into the cytosol and triggers activation of the cGAS‐STING pathway, which in turn promotes type I IFN production necessary for efficient cross‐priming of antitumor T cells.^29^ Taken together, these findings highlight the significance of the CD47‐SIRPα signaling axis to tumor immune evasion, as this pathway not only limits innate immune clearance of tumor cells but also acts as a barrier to DC‐driven adaptive immunity to cancer as well.

### 3.2 The leukocyte immunoglobulin‐like receptor B family of phagocytosis inhibitors

Though the CD47‐SIRPα axis is the most extensively studied phagocytosis checkpoint to date, additional pathways influencing this process have also been uncovered in the last decade. While these pathways have primarily been studied in the context of macrophages, given the shared expression of certain phagocytic receptors between these cells and DC, it is likely that tumoral influences on these signaling systems contribute to alterations in DC function as well. One such pathway that has been shown to impair macrophage‐mediated phagocytosis of tumor cells involves MHC class I signaling through the leukocyte immunoglobulin‐like receptor family member leukocyte immunoglobulin‐like receptor B1 (LILRB1). Specifically, inhibition of phagocytosis is driven by interaction between LILRB1 on macrophages and the MHC class I‐associated β2M subunit expressed by tumor cells,^30^ highlighting the potential universality of this innate checkpoint as a means for tumor immune evasion in cancer patients regardless of their HLA haplotype. Although tumor cell loss of MHC class I expression is a well‐described mechanism of immune escape, the selective pressure for such downregulation is applied only in the face of an effective CTL response, and tumor cell maintenance of MHC class I and its subversion of innate immune recognition and phagocytosis may actually explain the poor immunogenicity of many cancers. In this regard, LILRB1 expression is not restricted to macrophages – it is also expressed on DC, and its engagement on these cells is therefore likely to interfere with tumor uptake and immune stimulation by this innate population as well. Indeed, work in nontumor models has shown that LILRB1 signaling in DC inhibits Ca^++^ flux shortly after stimulation and impairs IL‐12 production and T cell activation by these cells.^31^, ^32^ Though the mechanism for LILRB1‐mediated inhibition of DC function has not been thoroughly investigated in fully differentiated DC, it is interesting that engagement of LILRB1 during DC differentiation from monocytic precursors led to retention of NF‐κB in the cytosol via an ABIN1/TINP1‐dependent mechanism. This interference with NF‐κB nuclear translocation led to impaired phagocytosis, decreased expression of MHC class I and II molecules, and reduced secretion of IL‐12 and IFN‐α by the resulting DC, which were poor stimulators of T cells.^33^ As tumors are frequently infiltrated by myeloid precursor populations, this pathway may be particularly relevant to the development of poorly immunogenic DC within the TME. With evidence accumulating that other members of the LILRB family also act as negative regulators of DC function,^34^ it will be important going forward to investigate how each of these family members impacts DC activity in the context of cancer, as these receptors may represent multiple targets for therapeutic interventions aiming to prevent tumor subversion of DC‐mediated immunity.

### 3.3 The CD24/Siglec‐10/G axis and other sialoglycan/siglec receptor interactions

The linkage of sialic acids to glycoproteins/glycolipids on the surface of mammalian cells is a distinguishing feature of the host that is often used by innate immune cells to differentiate self from non‐self. As such, the presence of sialoglycans on self cells can inhibit the activation of innate immune cells when recognized by members of the sialic acid‐binding immunoglobulin‐like lectin (Siglec) family of receptors.^35^ In this regard, the most recently discovered “don't eat me” signal found to confer tumor cell resistance to phagocytosis is CD24, a heavily glycosylated cell surface protein known as heat stable antigen. Weissman and colleagues reported upregulation of *CD24* gene expression in nearly all tumor types analyzed from TARGET and TCGA datasets and found that CD24 expression on breast and ovarian cancer cells inhibited phagocytosis by macrophages through engagement of Siglec‐10.^36^ Though the impact of tumor cell‐associated CD24 on DC was not investigated in this study, previous work has shown that the CD24/Siglec‐10/G axis limits DC responsiveness to damage‐associated molecular patterns (DAMPs) including HMGB1, HSP70, and HSP90.^37^ Similar to the effect of LILRB1 signaling in DC described above, the negative regulation of DC responsiveness to DAMPs by CD24/Siglec‐10/G signaling was associated with cytoplasmic retention of NF‐κB. Based on CD24's inhibition of macrophage function in the context of cancer and the inhibitory signaling mediated via Siglec‐10/G that can occur in DC, it will be important going forward to explore how the CD24/Siglec‐10/G axis might disrupt DC function within the TME, as this pathway might also serve as a significant barrier to the induction and/or maintenance of antitumor T cell responses by DC.

In addition to Siglec‐10/G, DC express a number of other inhibitory Siglecs, all of which share ITIM or ITIM‐like cytoplasmic motifs that drive negative regulatory signaling, typically via the SHP‐1 or SHP‐2 phosphatases.^38^ These receptors may also be particularly relevant to DC function within the TME. For instance, Siglec‐9 engagement by a cancer‐specific MUC1 mucin with sialylated O‐linked glycans during DC differentiation from monocytic precursors has been shown to impair costimulatory molecule expression by the resulting DC.^39^ Other tumor‐derived gangliosides have also been shown to impair DC differentiation and survival,^40^, ^41^, ^42^, ^43^ though particular Siglec receptor/ganglioside interactions were not evaluated in these earlier studies. Based on the diverse pattern of Siglec receptor expression on DC and the production of elevated levels or unique forms of cell‐bound and soluble glycans by cancer cells during tumor progression,^44^ a systematic evaluation of specific sialoglycan‐Siglec interactions and how they influence DC function in the TME may ultimately prove useful in the development of novel agents that aim to disrupt negative signaling pathways in DC and in turn enhance the quality of DC‐mediated antitumor immune responses.

### 3.4 CTLA‐4 and PD‐1: More than immune checkpoints for T lymphocytes

CTLA‐4 and PD‐1, the most‐well‐characterized immune checkpoints to date, have been studied extensively as negative regulators of T lymphocyte activation (see below). Recently, each of these immunoglobulin superfamily members has also been shown to negatively regulate the activity of DC through mechanisms driven by tumor cells as well as other cell populations frequently enriched in the TME. Regulatory T cells (Tregs), for example, can limit costimulatory molecule expression by DC through two distinct CTLA‐4‐mediated mechanisms: (a) transendocytosis and degradation of CD80/CD86^45^, ^46^ and (b) suppression of DC maturation by reverse signaling through CTLA‐4′s high affinity CD80 ligand.^47^ CTLA‐4 is also expressed on multiple tumor cell types,^48^ and breast cancer cell‐associated CTLA‐4 was found to suppress DC maturation, most likely through its ability to promote ERK and STAT3 signaling in these cells.^49^ Finally, DC themselves can express CTLA‐4 and are subject to both cell‐intrinsic and cell‐extrinsic mechanisms of suppression mediated by this immune checkpoint. Halpert *et al* recently found that intracellular CTLA‐4 stores were secreted by DC as part of microvesicles that could in turn interact with costimulatory molecules on bystander DC and trigger vesicular uptake, leading to loss of CD80/CD86 expression on the recipient DC.^50^ Whether such a mechanism occurs within DC in the TME is currently unknown, but the authors of this study did find that silencing CTLA‐4 in bone marrow‐derived DC (BMDC) prior to electroporation with B16 melanoma mRNA improved the antitumor efficacy of BMDC vaccination against established tumors. While this effect might have been due to the prevention of microvesicle‐mediated endocytosis of costimulatory molecules as described in their in vitro experiments, it is also possible that silencing CTLA‐4 in BMDC prevented intrinsic inhibitory signaling through this receptor, a phenomenon that has been demonstrated in human monocyte‐derived DC that display impaired T cell stimulatory activity and skewed IL‐10^high^/IL‐12^low^ secretion ratios following CTLA‐4 ligation.^51^, ^52^ Regardless of the mechanism(s) by which CTLA‐4 regulates DC function, these data highlight this inhibitory receptor as a relevant innate checkpoint in DC that can be targeted to enhance the immunostimulatory activity of these cells in the context of cancer.

Like CTLA‐4, PD‐1 has primarily been viewed as a critical immune checkpoint in T lymphocytes. However, PD‐1 is also expressed by innate immune cell populations and can negatively regulate their function as well. Indeed, the PD‐1/PD‐L1 axis has been shown to function as another phagocytic checkpoint in tumor‐associated macrophages. In another recent study by Weissman's group, it was demonstrated that whereas PD‐1‐deficient macrophages engulf PD‐L1^+^ and PD‐L1 KO CT26 colorectal cancer cells equally well, PD‐1^+^ macrophages engulfed PD‐L1 KO tumor cells significantly better that PD‐LI‐expressing tumor cells.^53^ How PD‐1 expression on DC might impact tumor cell phagocytosis has not been studied to date, but it is known that PD‐1 compromises the immunostimulatory function of DC. First reported in an *L. monocytogenes* bacterial infection model, it was found that PD‐1 expression by splenic DC limits production of the proinflammatory cytokines IL‐12 and TNF‐α during ex vivo restimulation, a phenomenon that could be reversed by addition of PD‐L1 blocking Ab to splenic cultures.^54^ In the context of cancer, immunosuppressive PD‐1^+^ DC have been isolated from tumors and ascites of mice challenged with ID8 ovarian cancer cells, and ligation of PD‐1 on these DC suppressed NF‐κB activation, Ag presentation, costimulatory molecule expression, and proinflammatory cytokine secretion.^55^, ^56^ PD‐1‐expressing DC have also been recovered from tumors in a murine model of liver cancer, and a population of PD‐1^+^, CD3^−^, CD11c^+^ cells also likely to be DC were detected in tumor tissue of hepatocellular carcinoma patients.^57^ Though the impact of PD‐1 signaling in these endogenous TIDC was not evaluated in this study, the authors did demonstrate in the murine liver cancer model that intratumoral delivery of PD‐1 KO BMDC suppressed tumor growth more efficiently than PD‐1‐expressing BMDC, and the improved tumor control in PD‐1‐deficient DC recipients correlated with an increased frequency of tumor‐infiltrating CD8^+^ T cells expressing perforin and granzyme B. Together, these data highlight the immune limiting effect of PD‐1 on DC, and they reveal the PD‐1/PD‐L1 axis as a checkpoint that can be targeted in these cells to improve antitumor immunity.

### 3.5 T cell Ig and mucin domain containing molecule‐3

T cell Ig and mucin domain containing molecule‐3 (TIM‐3) was first identified as a negative regulator of T lymphocytes nearly two decades ago,^58^ but it is now known to be expressed on many immune cell populations and can alter the function of multiple DC subsets. TIM‐3 negatively regulates IFN‐α production by plasmacytoid DC (pDC), potentially by mediating lysosomal degradation of the IRF7 transcription factor.^59^ TIM‐3 also promotes inhibitory signaling in splenic DC and BMDC, as agonistic crosslinking anti‐Tim‐3 Ab suppresses IL‐12 secretion by these cells in response to LPS‐stimulation.^60^ Importantly, upregulation of TIM‐3 on TIDC has been reported in lung, bladder, and breast cancer patients,^61^, ^62^, ^63^ and multiple mechanisms of DC immunosuppression are mediated by TIM‐3 within the TME. In murine models of breast cancer, TIM‐3 expression on intratumoral cDC1 cells suppressed production of the chemokine CXCL9, a known chemoattractant for CXCR3^+^ T lymphocytes, and the antitumor efficacy mediated by blocking either TIM‐3 or its ligand galectin‐9 was both CD8^+^ T cell‐ and CXCR3‐dependent,^63^ suggesting a suppressive role for TIM‐3 in TIDC‐mediated recruitment of T cells into tumor tissue. Additionally, Chiba *et al* reported a galectin‐9‐independent mechanism by which DC‐associated TIM‐3 suppresses antitumor immunity. In their study, interaction between HMGB1 and TIM‐3 on DC interfered with nucleic acid recruitment to endosomal compartments and impaired innate immune sensing of nucleic acids released from dying tumor cells.^61^ Based on HMGB1's known role as an alarmin that can induce danger signaling through RAGE and various TLR, it is interesting to speculate that TIM‐3‐mediated sequestration of HMGB1 might also prevent direct immunostimulatory signaling from this molecule in tumor‐associated DC. Though this possibility has yet to be investigated experimentally, it is clear that there are diverse immune regulatory effects mediated by TIM‐3 in tumor‐associated DC, and these pathways, as well as factors that drive TIM‐3 expression by DC, offer multiple points for therapeutic intervention to relieve suppression of these cells within the TME.

### 3.6 Other inhibitory receptors that negatively regulate tumor‐associated DC

A number of other inhibitory receptors that limit the function of DC are known to exist. Though less is known about the role of these receptors in the context of the TME as compared to the innate checkpoints described above, there is evidence that these other receptors can also be co‐opted by tumors as a means of subverting DC‐mediated immunity. The TAM receptor tyrosine kinases (RTK) are a family of receptors that include Tyro3, Axl, and Mer, all of which function to restrain inflammatory responses following phagocytic clearance of dying cells.^64^ These receptors bind two primary ligands complexed to phosphatidylserine on the surface of apoptotic cells, PROS1 and Gas6, both of which are expressed at high levels in many cancers.^65^, ^66^ Upon engagement on BMDC and monocyte‐derived DC, TAM receptors dampen inflammatory responses by upregulating expression of the SOCS1 and SOCS3 suppressors of cytokine signaling and by inhibiting the PI3K/Akt/NF‐κB pathway, respectively.^67^, ^68^ Though these specific mechanisms have not yet been demonstrated for TIDC in vivo, a recent study did demonstrate that TAM RTK signaling in DC negatively affects the accumulation of these cells within tumors, and a pan‐TAM RTK inhibitor directly enhanced MHC II expression on intratumoral DC and led to improved immunologic control of established tumors.^69^

Another inhibitory receptor, B‐ and T‐lymphocyte attenuator (BTLA), was found to be upregulated on DC from tumor tissue (as compared to those from normal adjacent tissue) of bladder cancer patients, and in vitro studies demonstrated that the BTLA ligand HVEM suppressed proinflammatory cytokine secretion by both cDC1 and pDC obtained from PBMC of healthy donors.^62^ Others have shown that the HVEM‐BTLA pathway is also critical for maintaining DC homeostasis,^70^ suggesting that BTLA may limit not only the activation but also the expansion of tumor‐associated DC.

Tumor‐infiltrating DC, as well as other myeloid populations, can also express high levels of V‐domain Ig suppressor of T‐cell activation (VISTA), a negative immune regulator that shares some structural features with PD‐L1. Mechanistically, VISTA restrains TLR/MyD88‐mediated signaling in several TIDC subsets and renders these cells tolerogenic.^71^ Though a ligand for VISTA has yet to be identified, antibody blockade of this regulatory protein has been shown to enhance the immunostimulatory functions of TIDC and to improve T cell effector function and tumor control in murine models of melanoma and bladder cancer.^72^ Immunohistochemial analyses of primary cutaneous melanomas have also shown significant correlation between VISTA expression and myeloid cell infiltrates, with VISTA expression being a poor prognostic indicator of disease‐specific survival.^73^ Interfering with VISTA signaling in TIDC may therefore be an effective strategy for improving tumor immunity and clinical outcome in cancer patients as well.

**FIGURE 1:**
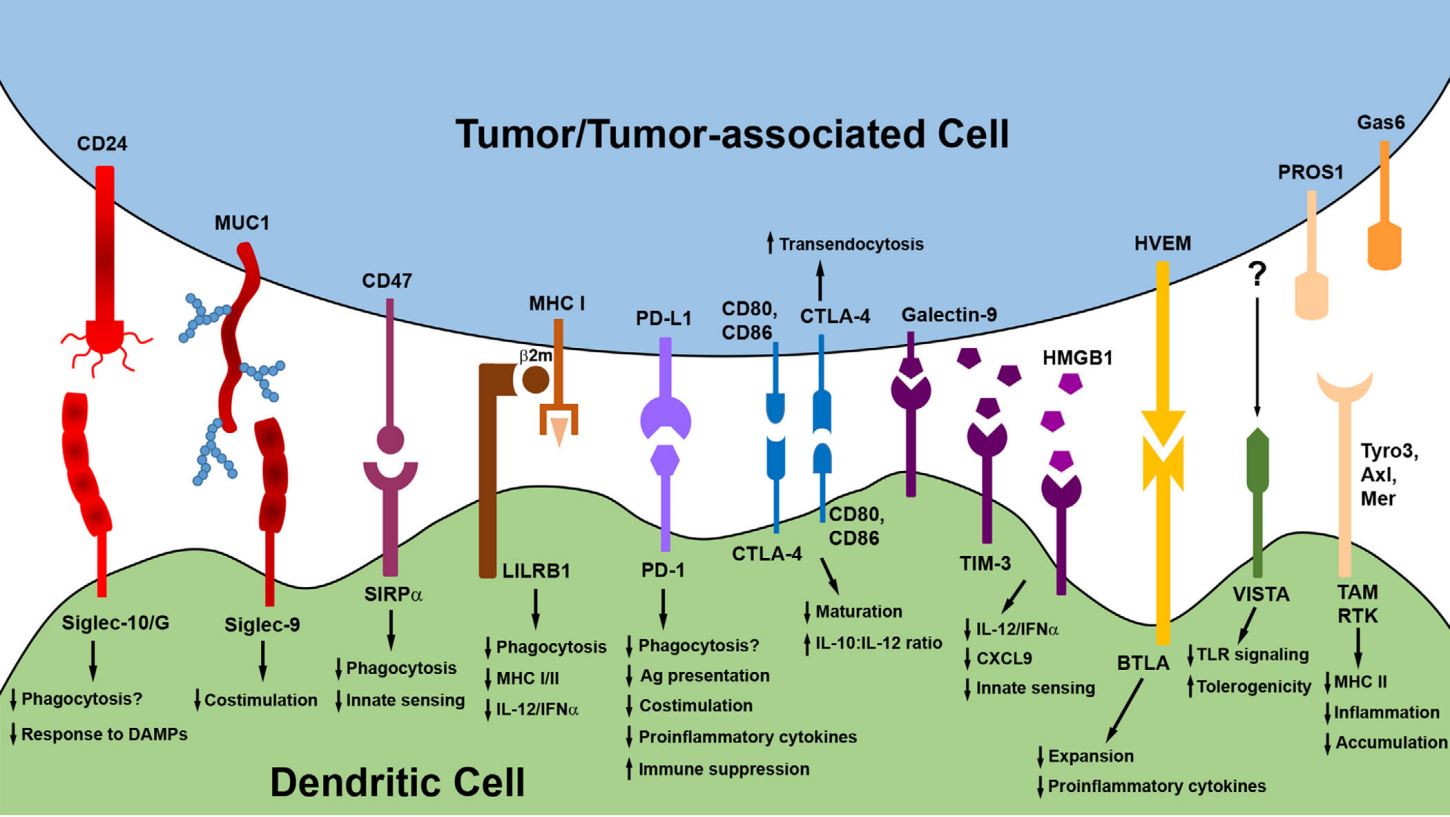
Innate immune checkpoints influencing DC function in the TME. A variety of innate immune checkpoints may limit DC function within the TME when engaged by ligands expressed on or secreted by tumor cells or other tumor‐associated populations, such as Tregs. Signaling through these innate checkpoint receptors compromises several DC functions that are critical to the induction, stimulation, and maintenance of antitumor immune responses. Mechanisms indicated with “?” represent those that have yet to be directly demonstrated in DC but that have been identified as outcomes of checkpoint receptor engagement in other innate populations (ie, macrophages) regulated by the same checkpoint pathway. Many of these checkpoint pathways are now targets for therapeutic interventions that aim to enhance the antitumor immune functions of DC, as described in detail in the main text

Together, these data highlight the diversity of inhibitory receptors expressed by DC and, in turn, the complexity of DC immunoregulation that ultimately occurs in the TME. As the expression and function of these and other innate checkpoints in DC are more fully elucidated within the context of the TME (Figure 1), new targets for personalized cancer therapies will surely emerge, and combinatorial regimens that neutralize multiple inhibitory pathways in DC are likely to become the most effective means of reversing/preventing tumor‐associated dysfunction in these cells. Such approaches have the potential to significantly improve the efficacy of DC‐mediated antitumor immune responses going forward.

## 4 METABOLIC CHECKPOINTS INFLUENCING DC FUNCTION IN THE TUMOR MICROENVIRONMENT

**FIGURE 2:**
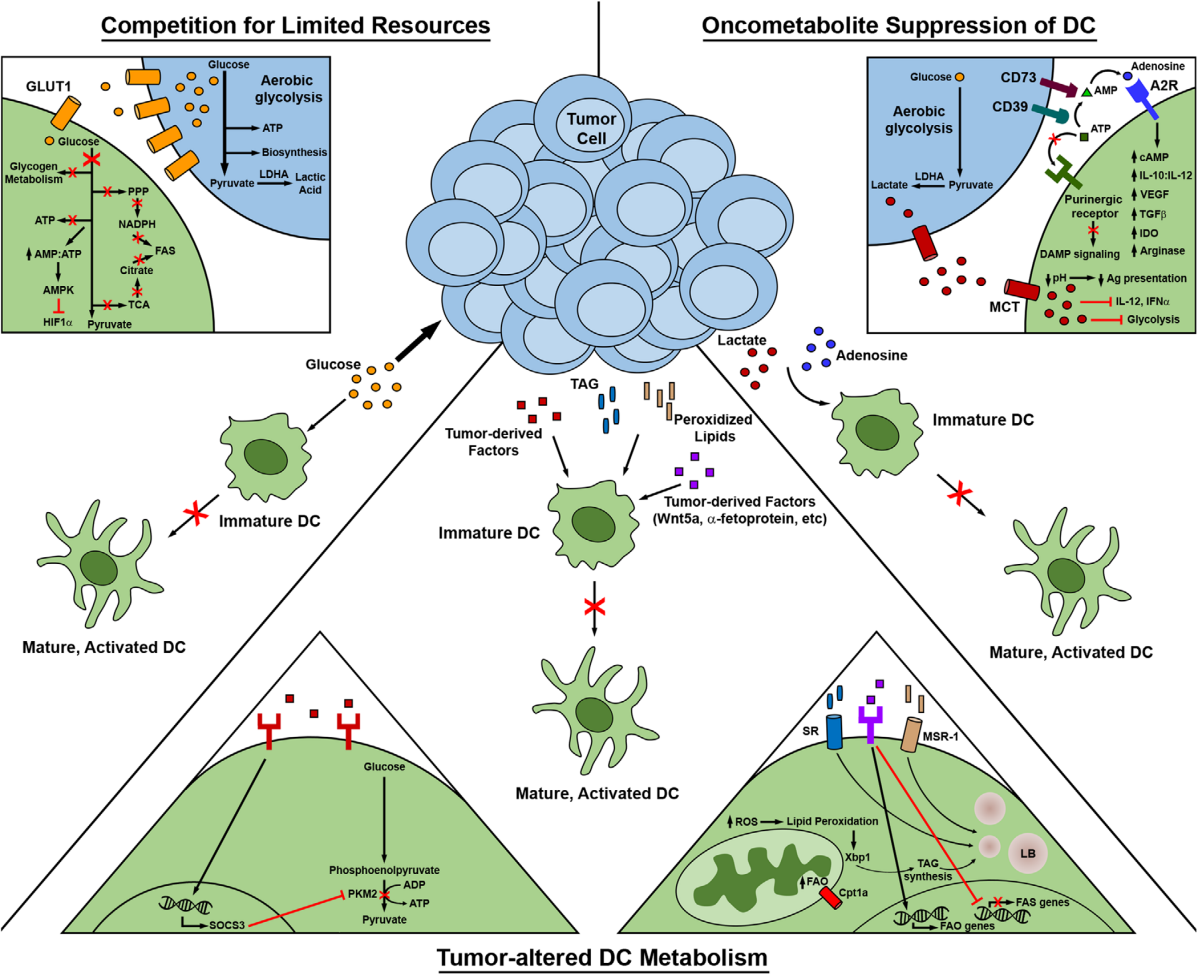
Metabolic regulation of DC function within the TME. DC metabolism, and in turn function, may be compromised within the TME in a number of ways. Tumor cells may outcompete DC for limited resources necessary to support metabolic function, tumor‐derived factors and metabolic products may directly alter metabolism within DC, and immunosuppressive oncometabolites generated by tumor cells may trigger a shift in DC from antitumor to pro‐tumor functionality. Therapeutic strategies that disrupt these mechanisms by which tumors regulate DC metabolism can restore the immunostimulatory and antitumor activity of DC, as described in detail in the main text. Abbreviations in this figure not defined in the main body of the text: TAG, triacylglycerol; SR, scavenger receptor; LB, lipid body; A2R, A2 adenosine receptor (A_2A_/A_2B_ receptors)

In addition to engaging the aforementioned innate checkpoints that negatively regulate DC function, both tumor and tumor‐associated cells also release a variety of factors that can either interfere with DC recruitment to tumor tissue or disrupt DC differentiation, maturation, and activation within the TME. While a number of immunosuppressive factors (TGF‐β1, IL‐10, VEGF‐A, etc) frequently enriched in the TME have recently been reviewed elsewhere,^8^, ^74^, ^75^ metabolically suppressive factors within the TME have also emerged as critical regulators of DC function (Figure 2). Though the discovery of these metabolic checkpoints has added increasing complexity to the various mechanisms of DC immunoregulation within the TME, insight into metabolic suppression of DC has also significantly expanded the repertoire of potential therapeutic targets that otherwise limit the immunostimulatory capacity of these cells in the context of cancer.

### 4.1 Tumor‐imposed limitations on DC carbohydrate/energy metabolism

The diverse functions of DC are tightly linked to the maturation and activation status of these cells. In recent years, it has become apparent that the phenotypic and functional plasticity of DC during their progression from an immature to a mature, activated state is driven by metabolic plasticity designed to support the specific functions of these cells. Immature DC fulfill their bioenergetic demands by generating ATP through oxidative phosphorylation (OXPHOS), and this process is driven primarily by fatty acid oxidation (FAO),^76^ consistent with the need of these highly phagocytic cells to catabolize fatty acid substrates generated from lipolysis of apoptotic cell membranes acquired during engulfment. Shortly after stimulation, DC maintain OXPHOS activity to generate ATP but undergo a metabolic shift toward glycolysis in order to yield substrates to support the tricarboxylic acid (TCA) cycle and pentose‐phosphate pathway (PPP), which yield citrate and NADPH for the de novo lipogenesis needed to support expansion of endoplasmic reticulum (ER) and Golgi apparatus membrane mass.^77^ It is believed that the dedication of resources toward these organelles supports the protein synthesis and trafficking needs of DC that are transitioning from phagocytic cells to functional APC that express/secrete newly synthesized cell surface costimulatory molecules and immunostimulatory cytokines. It has also been shown that early glycolytic flux is required for CCR7 oligomerization and DC migration to draining lymph nodes,^78^ where mature, activated DC typically prime T cell responses. Ultimately, genetic reprogramming of activated DC triggers a long‐term commitment to glycolysis to fuel ATP production in the face of the lost mitochondrial respiratory function that accompanies nitric oxide or type I IFN production by these cells.^79^, ^80^ Interestingly, recent metabolic tracing studies demonstrated that the glycolytic switch in newly activated DC relies heavily on the metabolism of glycogen. This work supports a model in which intracellular glycogen stores present in DC prior to activation drive the early glycolytic burst of these cells following stimulation. Additionally, extracellular glucose taken up by DC following glycolytic reprogramming is utilized not only for direct catabolism but also for rerouting into a pathway of glycogen synthesis‐glycogenolysis known as the glycogen shunt.^81^ Though the functional outcomes of these distinct pathways for extracellular glucose utilization by DC during activation remain to be explored, it is clear that both glucose and glycogen metabolism are critical to achieving immunostimulatory functions of DC.

In order to support the biosynthetic and energy demands of rapid cell division, tumor cells also frequently undergo rewiring to a primarily glycolytic mode of metabolism, not only in hypoxic environments where OXPHOS is naturally limited but also under normoxic conditions, a phenomenon known as the Warburg effect (aerobic glycolysis). While the aforementioned studies on glycolytic switching in activated DC have primarily been performed on BMDC and monocyte‐derived DC in vitro (due to technical limitations that preclude analysis of this process in DC in vivo), there are a number of potential and documented effects of glycolytically active tumor cells on DC function within the TME. First, competition between tumor cells and DC for limited resources may simply restrict glucose availability to TIDC. In this regard, a recent comparison of progressor versus regressor tumors revealed diminished glucose concentration in the extracellular milieu of progressor tumors,^82^ suggesting that glucose depletion in the TME may limit DC uptake of extracellular glucose needed to sustain a glycolytic switch. As long‐term commitment to glycolysis is needed to support ATP production in activated DC, a failure to maintain glycolytic activity is likely to lead to elevated AMP:ATP ratios in TIDC, an outcome that could in turn trigger AMPK‐mediated downregulation of HIF‐1α,^83^, ^84^ a transcriptional regulator of several enzymes essential to glycolytic metabolism.^85^ HIF‐1α also regulates expression of enzymes necessary for glycogen synthesis,^86^ suggesting that interference with the aforementioned glycogen shunt for any glucose that is acquired by DC in the TME may also impair the metabolic and immunologic functions of these cells, a possibility that remains to be investigated. In addition to these passive mechanisms of tumor‐altered carbohydrate metabolism by DC that could result from glucose depletion in the TME, multiple tumor types have been shown to actively inhibit glycolysis in these cells as well. In murine models of melanoma as well as lung and ovarian cancers, tumor‐associated DC exhibited increased expression of SOCS3, which was found to suppress activity of the pyruvate kinase M2 (PKM2) enzyme responsible for catalyzing the final step of glycolysis.^87^ In this study, SOCS3 was also found to limit the PKM2‐driven antitumor efficacy of a DC‐based vaccine against established LLC tumors. Together, these data suggest that tumor‐associated regulators of glycolytic metabolism in DC could therefore be useful targets for therapies relying on the activity of either endogenous or exogenous DC.

### 4.2 Tumor‐altered lipid metabolism in DC

As alluded to above, de novo lipogenesis plays a key role in supporting membrane mass for increased ER and Golgi activity during DC maturation and activation. At the same time, lipid accumulation in tumor‐associated DC is a known hallmark of immune dysfunction in these cells. This apparent dichotomy may be explained by the nature of lipid content found in appropriately activated versus tumor‐altered DC, the latter of which could be compromised by either: (a) tumor‐associated suppression of lipid synthesis needed to support organelle membrane mass and vesicular transport of molecules for T cell stimulation or (b) tumor‐induced uptake/generation of lipids detrimental to DC function. In support of the former, tumor‐derived α‐fetoprotein has been shown to suppress expression of several genes involved in fatty acid synthesis (FAS) in DC,^88^ and the defects in differentiation and T cell stimulatory capacity of these cells^89^ may therefore ultimately arise from limitations in lipid‐mediated trafficking of proteins otherwise needed for potent APC activity. On the other hand, DC functionality can also be compromised by the uptake of lipids enriched within the TME, a process that can be influenced by lipid metabolism in tumor cells themselves. In a murine model of ovarian cancer, fatty acid synthase (FASN) expression in tumor cells correlates with TME lipid content, including both saturated and unsaturated fatty acids as well as triacylglycerols, and TIDC isolated from tumors with high FASN expression exhibit elevated lipid levels and poor T cell stimulating activity when compared to those recovered from tumors in which FASN is silenced. Importantly, lipid accumulation and DC dysfunction in this model could be reversed therapeutically by treatment with a FASN inhibitor,^90^ demonstrating the potential of metabolic interventions to support the antitumor immune functions of DC.

In addition to increasing the lipid content available to DC within the TME, tumors can also actively promote lipid acquisition by DC by inducing their expression of scavenger receptors such as MSR‐1.^91^ In particular, the uptake and accumulation of peroxidized lipids in TIDC has been found to interfere with Ag cross‐presentation through a mechanism mediated by lipid bodies containing oxidatively truncated lipids covalently bound to the HSP70 chaperone protein, an interaction that prevents peptide:MHC complex trafficking from late endosomes/lysosomes to the cell surface.^92^, ^93^ Elevated reactive oxygen species (ROS) in tumor‐associated DC also contribute to intracellular lipid peroxidation, leading to activation of the ER stress sensor XBP1 that in turn drives triglyceride biosynthesis and further accumulation of lipids that blunt Ag presentation.^94^ Beyond these lipid‐associated defects in Ag presentation, DC maturation and proinflammatory cytokine secretion have also been found to be negatively influenced by oxidized fatty acids and polyunsaturated fatty acids, respectively.^95^, ^96^ Finally, the Wnt‐β‐catenin pathway has been shown to drive the oxidation of fatty acids in tolerogenic tumor‐associated DC that not only fail to support CD8^+^ T cell activation but that also promote Treg induction.^97^, ^98^ Mechanistically, tumor‐derived Wnt5a activates β‐catenin in DC, leading to PPAR‐γ‐mediated induction of the CPT1A mitochondrial fatty acid transporter and a shift to FAO metabolism. FAO in turn suppresses expression of IL‐6 and IL‐12, and diversion of TCA products to the heme biosynthesis pathway leads to increased production of the protoporphyrin IX prosthetic group necessary for full enzymatic activity of indoleamine 2,3‐dioxygenase‐1 (IDO1), a potent inducer of Tregs.^98^ Although these studies collectively highlight diverse mechanisms by which altered lipid metabolism compromises the function of tumor‐associated DC, they also reveal a number of therapeutic strategies for regulating fatty acid metabolic programs in both DC and tumor cells that have the potential to significantly enhance DC function in the context of cancer.

### 4.3 Suppression of DC by tumor‐derived metabolites

In addition to limiting the availability of nutrients and other key resources for use by immune cell populations infiltrating the TME, the extreme metabolic demands of rapidly growing tumor cells also result in the accumulation of toxic by‐products that are detrimental to immune function. In this regard, another consequence of the glycolytic switch that often occurs in tumor cells is TME accumulation of lactic acid, an oncometabolite that impairs DC function in a variety of ways. Lactate‐driven DC dysfunction was first described in monocyte‐derived DC differentiated in the context of tumor spheroids, where tumor suppression of IL‐12 secretion by DC could be reversed by blocking lactic acid production with a lactate dehydrogenase A (LDHA) inhibitor.^99^ Likewise, LDHA inhibition of ex vivo‐cultured lung tumor cells reduced the suppressive effects of tumor‐conditioned media on IFN‐α production by Flt3L‐differentiated BMDC.^100^ This study also found that lactic acid triggered endosomal acidification and Ag degradation in DC and reduced their ability to prime CD8^+^ T cells and confer antitumor immune protection in vivo. Most recently, lactic acid was also found to suppress IFN‐α production by pDC via two distinct mechanisms. First, lactate signaling through the GPR81 receptor on pDC mobilized intracellular Ca^++^ stores, which in turn dampened IFN‐α induction through activation of the phosphatase calcineurin. Second, lactic acid import into pDC through monocarboxylate transporters (MCT) increased intracellular lactate and suppressed the glycolytic switch needed to induce efficient IFN‐α expression.^101^ That intratumoral pDC were found to express higher levels of both GPR81 and MCT than their circulating counterparts underscores the significance of these mechanisms for compromising pDC function within the TME. Importantly, in addition to the potential consequences of reduced IFN‐α expression on the maintenance of CTL effector function within the TME, it was found that lactate conditioning of pDC also reprograms these cells toward increased tryptophan metabolism, a pathway that promotes Treg induction through release of kynurenine,^101^ as discussed in more detail below.

Another oncometabolite that has adverse consequences for antitumor immunity in the TME is adenosine, which often accumulates to high levels as a result of the metabolism of extracellular ATP. Extracellular ATP itself is frequently elevated in the TME,^102^ and while its release from tumor cells can promote immunogenic signaling as a DAMP through purinergic receptors on DC,^103^ it is often converted into adenosine by the ectonucleotidases CD39 and CD73,^104^, ^105^ leading instead to immunosuppressive signaling through A_2A_ and A_2B_ adenosine receptors on various immune cell populations. In addition to its well‐documented role in the regulation of T cell responses (see below), adenosine has also been shown to repress the immunostimulatory functions of DC. In a murine melanoma model, tumor‐associated DC function was improved by deletion of the A_2A_ receptor on DC, which resulted in significantly reduced *Il10* gene expression and slightly improved IL‐12 production by these cells,^106^ a finding consistent with data from in vitro studies evaluating the effects of adenosine on human monocyte‐derived DC.^107^ Adenosine signaling through the A_2B_ receptor has also been shown to drive gene expression for several tumor‐promoting factors (VEGF, TGF‐β, IDO, arginase, etc) in DC, and intratumoral injection of adenosine‐conditioned DC was found to enhance the vascularization and growth of LLC tumors.^108^ Mechanistically, adenosine signaling promotes accumulation of cAMP in DC, leading to activation of PKA and Epac pathways that polarize these cells to a tumor‐promoting phenotype (IL‐10^high^/IL‐12^low^) by increasing expression of NF‐κB pathway regulators.^109^ Together, these studies reveal a number of possible strategies for interfering with adenosine‐mediated suppression of DC in the TME, some of which have already shown efficacy in preclinical systems. These approaches include targeting the CD39/CD73 ectonucleotidases that yield adenosine from ATP, blocking the expression or activity of A_2A_/A_2B_ adenosine receptors on DC, and inhibiting intracellular signaling components that mediate the suppressive effects of adenosine on DC.^106^, ^110^, ^111^

## 5 IMMUNE CHECKPOINTS INFLUENCING T CELL FUNCTION IN THE TUMOR MICROENVIRONMENT

Similar to DC, T lymphocytes also express a number of receptors that function to restrict aberrant immune reactivity. While necessary to prevent autoimmunity and hyperactive responses to acute infections that are cleared quickly, engagement of these adaptive checkpoints in the context of cancer can lead to tumor immune escape by limiting the duration and quality of antitumor T cell responses. Insight into the most well‐studied of these negative regulatory pathways has paved the way for immune checkpoint blockade (ICB), a therapeutic approach to “release the brakes” on the immune system that has achieved unprecedented clinical success against many cancer types.^1^ With the discovery of new T lymphocyte checkpoints in recent years and an improved understanding of the mechanisms underlying tumor cell resistance to the first generation of immune checkpoint inhibitors, there is great promise for next‐generation and combinatorial checkpoint blockade therapies to unleash even more potent antitumor T cell responses and further improve patient response to ICB therapy in the future.

### 5.1 CTLA‐4

The first immune checkpoint found to negatively regulate the function of T lymphocytes was the co‐inhibitory receptor CTLA‐4.^112^ Upregulated on T cells shortly after stimulation, CTLA‐4 binds to the same CD80/CD86 ligands as the CD28 costimulatory receptor, but with higher affinity. In addition to limiting costimulation by outcompeting CD28 for ligand binding, CTLA‐4 engagement also transmits inhibitory signals to T lymphocytes, with cell type‐specific consequences. In CD4^+^ T cells, CTLA‐4 engagement limits T cell activation and differentiation during the priming phase of a response, ultimately inducing anergy.^113^, ^114^, ^115^, ^116^ In CD8^+^ T cells, on the other hand, CTLA‐4 does not impair priming but instead regulates the magnitude of recall responses to secondary stimulation,^117^, ^118^ which could limit the efficacy of both natural and adoptively transferred CTL following Ag re‐exposure within the TME. Indeed, it was recently reported that TGF‐β‐driven CD80 expression on tumor‐initiating stem cells promotes resistance to adoptive cell transfer (ACT) therapy via a CTLA‐4‐dependent mechanism.^119^ Though it remains to be explored, such a process means that lymph node‐invasive tumor cells, in addition to cross‐presenting APC, might also limit the priming of naïve helper T cell responses following direct presentation of tumor Ag within draining lymph nodes. Finally, CTLA‐4 is critical to the immunosuppressive activity of Tregs, driving transendocytosis of costimulatory molecules expressed on DC and thereby limiting their capacity to support the activation of both CD4^+^ and CD8^+^ T cells.^45^, ^46^

Following preclinical studies demonstrating that CTLA‐4 blockade diminishes Treg activity and restores antitumor T cell effector function,^120^ a subsequent clinical trial showing the therapeutic efficacy of an anti‐CTLA‐4 antibody in melanoma patients^121^ led to FDA approval of ipilimumab as the first immune checkpoint inhibitor for cancer treatment. In both murine models and cancer patients, anti‐CTLA‐4 monotherapy is associated with an expansion of ICOS^+^ CD4^+^ Th1 effectors.^122^, ^123^ A concomitant increase in CD8^+^ T cells with an exhausted phenotype is also observed following CTLA‐4 blockade, and combinatorial inhibition of other immune checkpoints (see below) can harness the potential of this expanded population for improved tumor immune control. As such, ipilimumab has since been approved in combination with nivolumab (anti‐PD‐1) therapy for certain cases of melanoma, renal cell carcinoma, hepatocellular carcinoma, and microsatellite instability‐high or mismatch repair deficient colorectal cancer. Another CTLA‐4 checkpoint inhibitor, tremelimumab, is also currently under evaluation as part of combinatorial regimens for various other malignancies. Though both ipilimumab and tremelimumab have been shown not to deplete FOXP3^+^ Tregs in cancer patients, it is possible that modification of the Fc regions of these antibodies may confer this ability shared by antibodies against murine CTLA‐4 and further improve the therapeutic benefit of CTLA‐4 blockade in humans.^124^

### 5.2 PD‐1

The early observation that CTLA‐4 blockade could enhance tumor Ag‐specific CD4^+^ T cell priming but could not overcome the eventual tolerization of these cells highlighted the contribution of alternative pathways to the regulation of T lymphocyte function.^125^ Indeed, several other co‐inhibitory checkpoints for T lymphocytes have now been identified, and of these, the PD‐1/PD‐L1 axis is the most‐well studied. PD‐1 is another member of the immunoglobulin superfamily and is upregulated on both CD4^+^ and CD8^+^ T lymphocytes shortly after stimulation.^126^ It shares two ligands, PD‐L1 and PD‐L2, the latter of which is expressed primarily on hematopoietic cell populations and the former of which is expressed on both hematopoietic cells as well as non‐hematopoietic cells from many tissue types.^127^ Upon engagement of PD‐1, both PD‐L1 and PD‐L2 have been shown to suppress T cell effector function,^128^, ^129^ though PD‐L2's role in immunosuppression is controversial, as it has also been shown to protect T cells from PD‐L1‐mediated suppression in certain contexts.^130^

The PD‐1/PD‐L1 axis negatively regulates T lymphocyte activity in a variety of ways. In addition to driving the differentiation and immunosuppressive activity of inducible Tregs,^131^ this pathway plays a significant role in the exhaustion of peripheral T cells during the effector phase of a response, particularly in cases of repeated Ag exposure, such as that encountered during chronic viral infection or in the context of progressing tumors.^122^, ^132^, ^133^, ^134^, ^135^ There is also accumulating evidence that PD‐1/PD‐L1 interaction during the induction phase of a T cell response can impact clonal expansion and effector cell differentiation as well.^136^, ^137^, ^138^ Indeed, PD‐L1 expressed on either tumor cells or tumor‐associated APC is sufficient to blunt antitumor T cell responses, with negative regulation of T cell function being mediated by PD‐L1 both within the TME and within tumor‐draining lymph nodes.^139^, ^140^, ^141^ Mechanistically, PD‐1 engagement by PD‐L1 promotes recruitment of the SHP‐2 phosphatase to the immunological synapse, leading to dephosphorylation of both TCR and CD28 signaling components and downregulation of the PKC and MAPK signaling pathways.^131^, ^142^, ^143^, ^144^, ^145^ PD‐1/PD‐L1 interaction also drives CD3 internalization and downregulation of the TCR,^146^ a phenomenon frequently observed in tumor‐infiltrating lymphocytes (TIL). Another consequence of PD‐1 engagement on T cells is its impact on metabolism, as PD‐1‐mediated inhibition of glycolysis and OXPHOS leads to bioenergetic insufficiencies that predispose T cells toward exhaustion.^147^, ^148^ Considering the constraints on T cell metabolism that are frequently encountered within the TME (described in detail below), this mechanism may be particularly relevant to tumor immune escape as it is likely to exacerbate metabolic deficiencies in tumor‐infiltrating T cells.

Like CTLA‐4 blockade, therapeutic targeting of the PD‐1/PD‐L1 pathway also enhances antitumor T cell reactivity, and immune checkpoint inhibitors against both PD‐1 (nivolumab, pembrolizumab, and cemiplimab) and PD‐L1 (atezolizumab, avelumab, and durvalumab) are now standard‐of‐care therapy for many cancer types.^1^ Unlike CTLA‐4 blockade, however, PD‐1 inhibitors do not enhance CD4^+^ effector T cell frequency within tumors but instead primarily drive expansion of phenotypically exhausted CD8^+^ T cells.^122^, ^149^ Though these expanded CD8^+^ T cells retain expression of cell surface markers indicative of exhaustion, likely due to epigenetic maintenance of co‐inhibitory molecule expression,^150^ the accumulation of less exhausted non‐terminally differentiated (PD‐1^+^ TIM3^low^ TBET^+^ EOMES^−^) and fully exhausted terminally differentiated (PD‐1^high^ TIM3^+^ TBET^+^ EOMES^+^) CD8^+^ T cells is associated with improved tumor control. These data suggest that PD‐1 inhibition may improve tumor immunity by temporarily reinvigorating the antitumor reactivity of cells that had already been rendered functionally exhausted and/or by enhancing the activity of partially exhausted cells and preventing their transition to a completely exhausted state.

Interestingly, while combinatorial blockade of both CTLA‐4 and PD‐1 has additive effects on the frequency of many of the specific T cell subsets stimulated by each monotherapy, dual CTLA‐4/PD‐1 blockade also elicits responses distinct from those achieved with monotherapy. Namely, combination therapy decreases the intratumoral frequency of phenotypically exhausted CD8^+^ T cells that are otherwise expanded by anti‐PD‐1 monotherapy and instead drives the accumulation of activated terminally differentiated effector CD8^+^ T cells within tumors.^123^ It was also recently shown that responders to dual CTLA‐4/PD‐1 checkpoint blockade are enriched for effector memory CD8^+^ T cells within tumor biopsies.^151^ The differential responses to combination therapy versus individual anti‐CTLA‐4 and anti‐PD‐1 monotherapies are in keeping with unique transcriptional signatures identified in CD4^+^ and CD8^+^ TIL following treatment with these regimens,^122^, ^152^, ^153^ and these data support combination therapy as the most effective means of programming T cells for robust antitumor reactivity. Indeed, recent clinical trials have highlighted the success of combination ICB therapy against CTLA‐4 and PD‐1. In the CheckMate067 trial, the 5‐year overall survival rate for advanced melanoma patients receiving combination ipilimumab + nivolumab as frontline therapy exceeded 50%, whereas this rate was 44% for patients on single‐agent nivolumab and only 26% for those on ipilimumab alone.^154^ Though not as dramatic, a significant improvement in patient survival was also reported for NSCLC patients treated with this same combination therapy versus nivolumab monotherapy or chemotherapy.^155^ Despite the promise of these and related trials, though, an ongoing challenge in the field is the need to better understand mechanisms of innate and acquired resistance to CTLA‐4, PD‐1, and PD‐L1 checkpoint blockade. To this end, significant efforts are now being focused on targeting additional regulatory pathways that are known to compromise the quality of antitumor T cell responses, as discussed in more detail below.

### 5.3 Lymphocyte activation gene 3

The observation that many patients do not respond to CTLA‐4 and PD‐1/PD‐L1 checkpoint blockade despite harboring TIL‐enriched tumors underscores the relevance of additional T cell‐regulating mechanisms within the TME. Indeed, tumor gene expression signatures from many anti‐CTLA‐4/anti‐PD‐1 non‐responders indicate that other co‐inhibitory receptors should also be evaluated when considering optimal combinatorial checkpoint blockade strategies.^151^ One such co‐inhibitor that is upregulated in a large percentage of patients who fail to respond to anti‐CTLA‐4/anti‐PD‐1 therapy is lymphocyte activation gene 3 (LAG‐3). Named as a result of its upregulation on activated T lymphocytes, LAG‐3 actually functions to suppress T cell activation, impairing cell cycle progression and expansion of both CD4^+^ and CD8^+^ T lymphocytes.^156^, ^157^ In the context of cancer, LAG‐3 has been shown to limit both the accumulation and effector function of CD8^+^ T cells in tumor tissue,^158^ and its elevated expression on tumor‐infiltrating Tregs correlates with enhanced immunosuppressive activity by these cells.^159^, ^160^

LAG‐3‐mediated suppression of T cell responses can be achieved in a number of ways. First, inhibitory signaling via LAG‐3 is transmitted by its cytoplasmic KIEELE domain when the receptor binds with high affinity to MHC class II,^161^ highlighting the potential for both tumor‐associated APC and MHC class II‐expressing cancer cells to suppress T cell function via this pathway. In addition to direct inhibitory signaling within T cells, LAG‐3/MHC class II interactions can also indirectly interfere with T cell activation by reverse signaling through the MHC, a mechanism that enables LAG‐3‐expressing Tregs to suppress DC maturation.^162^ Other LAG‐3 ligands can also negatively regulate T cell function within the TME. Galectin‐3 is a soluble galactoside‐binding lectin secreted by various tumor types that binds to LAG‐3 and suppresses antitumor CD8^+^ T cells.^163^ Similarly, the LSECTin lectin expressed on melanoma cells has also been shown to suppress antitumor T cell activity via a LAG‐3‐dependent mechanism.^164^

In keeping with the hypothesis that LAG‐3 may limit clinical responses to currently approved immune checkpoint inhibitors, co‐expression of LAG‐3 and PD‐1 was reported on both CD4^+^ and CD8^+^ TIL isolated from primary tumors of renal cell carcinoma patients, and when compared to PD‐1 blockade alone, dual blockade of both LAG‐3 and PD‐1 enhanced IFN‐γ production by these cells following ex vivo stimulation.^165^ Though the same benefit of dual LAG‐3/PD‐1 blockade over PD‐1 blockade alone was not observed in double positive tumor Ag‐specific CD8^+^ TIL isolated from ovarian cancer patients, it was achieved when both co‐inhibitors were targeted during the initial priming of Ag‐specific T cells isolated from PBL.^166^ Differences in these studies may reflect the extent to which TIL had become exhausted in each setting and/or expression of yet other co‐inhibitory receptors on ovarian cancer‐derived TIL that continued to repress T cell effector function despite dual LAG‐3/PD‐1 blockade. Still, both studies demonstrate that targeting LAG‐3 at various stages of an antitumor immune response does have the potential to improve tumor‐specific T cell reactivity. In this regard, work in the MC38 murine tumor model has demonstrated that LAG‐3 synergizes with PD‐1 in the suppression of antitumor T cell responses and that dual blockade is often curative for established tumors resistant to either monotherapy.^167^ It is also worth noting that a number of factors in the TME have now been implicated in the induction of LAG‐3 expression on T cells, including IL‐6, IL‐10, and tumor‐associated APC,^166^ and targeting these LAG‐3 inducers may also prevent suppression of antitumor T cell immunity by this checkpoint pathway.

### 5.4 T cell Ig and mucin domain containing molecule‐3

Another co‐inhibitory receptor frequently upregulated on exhausted TIL and intratumoral Tregs is TIM‐3.^168^, ^169^, ^170^ Though TIM‐3 has multiple ligands, most studies in tumor models and cancer patients to date have implicated galectin‐9 as the major trigger for TIM‐3‐mediated suppression of T cells.^171^, ^172^, ^173^ Current evidence supports a model whereby this suppression arises as Bat3 is released from TIM‐3′s cytoplasmic tail during receptor engagement by galectin‐9, thereby enabling inhibitory signaling that is otherwise repressed when Bat3 is bound.^174^ In this regard, it is worth noting that *Bat3* gene expression is significantly reduced in exhausted TIM‐3^+^ TIL isolated from murine mammary adenocarcinomas^174^ and that the long noncoding RNA lnc‐Tim3 binds to TIM‐3′s cytoplasmic tail and promotes release of Bat3 in exhausted CD8^+^ TIL from hepatocellular carcinoma patients.^175^ In addition, TIM‐3 is typically expressed at higher levels on intratumoral Tregs as compared to peripheral Tregs, and its upregulation is associated with more robust immunosuppressive activity.^168^, ^169^, ^176^, ^177^ Based on these findings, it is not surprising that TIM‐3 blockade has been found to augment antitumor immunity in multiple ways, with evidence for both enhanced CD8^+^ and CD4^+^ T cell effector activity and reduced Treg frequency emerging as mechanisms of therapeutic efficacy.^178^, ^179^

Considering TIM‐3′s role in inhibiting antitumor immune responses, it is worth noting that its co‐expression with PD‐1 marks CD8^+^ T cells that are more heavily exhausted than single‐positive PD‐1‐expressing cells.^174^, ^180^, ^181^, ^182^ Moreover, TIM‐3 expression on TIL is upregulated following PD‐1 blockade and has been linked with adaptive resistance to anti‐PD‐1 monotherapy,^183^, ^184^ suggesting that TIM‐3 might function as a failsafe to overcome loss of PD‐1‐mediated inhibition of T cell responses. Indeed, sequential or combinatorial blockade of TIM‐3 and PD‐1/PD‐L1 has shown enhanced antitumor efficacy in several murine models^172^, ^180^, ^183^, ^184^ and dual blockade of these checkpoints augments the effector activity of ex vivo‐stimulated CD8^+^ TIL from hepatocellular carcinoma patients.^185^ That similar data have also been reported for tumor‐bearing mice undergoing dual TIM‐3/CTLA‐4 blockade^178^ and for TIL treated with other combinations of checkpoint inhibitors^185^ underscores the potential utility of combinatorial approaches to checkpoint blockade therapy as a means of eliciting robust antitumor immunity.

### 5.5 TIGIT and related PVR/nectin family members

The T cell immunoreceptor with Ig and ITIM domains (TIGIT) is an inhibitory receptor belonging to the poliovirus receptor (PVR)/nectin family. Related members of this family include CD96, CD112R, and DNAM‐1 (CD226), which together with TIGIT comprise a complex immunoregulatory system for T lymphocytes and other immune cell populations. With respect to T cells, TIGIT can be highly expressed on Tregs and is upregulated on both activated CD4^+^ and CD8^+^ T lymphocytes, where it functions to dampen immune reactivity.^186^, ^187^ Its ligands include CD112, CD113, and CD155, many of which are expressed on various tumor types as well as tumor‐associated DC.^188^, ^189^, ^190^, ^191^ Though these ligands are shared between TIGIT and related PVR family members, their impact on T cell function is receptor‐dependent. For instance, while CD155 can costimulate T cells when signaling through DNAM‐1, such signaling is often limited by competition for ligand binding with the higher affinity TIGIT and CD96 receptors, both of which suppress T cells through their ITIM and other inhibitory signaling motifs.^192^, ^193^ Similarly, interference with CD112‐mediating signaling through DNAM‐1 has been reported for CD112R, which also has higher affinity for its shared ligand and, like TIGIT, transmits inhibitory signals to T cells.^194^ Finally, immunostimulatory DNAM‐1 signaling can also be compromised by a mechanism unrelated to ligand competition, as *cis* interaction with TIGIT has been shown to prevent the homodimerization necessary for DNAM‐1 function.^195^

In addition to its influence over DNAM‐1 signaling, TIGIT has been found to drive T cell dysfunction by a variety of cell‐intrinsic and cell‐extrinsic mechanisms. Inhibitory signaling through TIGIT has been shown to impact T cell metabolism, reprogramming cells away from the glycolytic flux needed to support T cell activation. When compared to TIGIT^−^ CD8^+^ T cells isolated from gastric cancer patients, functionally exhausted, glycolytically deficient TIGIT^+^ CD8^+^ T cells exhibited reduced expression of the GLUT1 glucose importer and the hexokinase 1/2 glycolysis‐initiating enzymes. Importantly, glycolytic metabolism could be restored in CD8^+^ T cells co‐cultured with gastric cancer cell lines when TIGIT‐CD155 interactions were disrupted.^196^ Cell‐intrinsic signaling through TIGIT also supports immunoregulatory functions of Tregs that correlate with the enhanced capacity of TIGIT^+^ Tregs to suppress T_H_1/T_H_17 differentiation.^197^ Moreover, elegant studies in which various combinations of TIGIT^+^ versus TIGIT‐deficient CD8^+^ T cells and Tregs were co‐transferred into *Rag* ^−/−^ mice prior to tumor challenge have also highlighted the significance of TIGIT signaling in Tregs to the suppression of CD8^+^ TIL effector function and overall antitumor immunity.^198^ Finally, with regard to its cell‐extrinsic functions, TIGIT can also drive reverse signaling through CD155, and TIGIT‐mediated signaling through this ligand on DC augments IL‐10/IL‐12p40 ratios and drives DC‐dependent inhibition of T cell activation.^199^

TIGIT expression has been reported on T cells isolated from tumor tissue of a variety of cancer types, including follicular lymphoma, multiple myeloma, melanoma, gastric cancer, NSCLC, and HNSCC, among others.^191^, ^195^, ^200^, ^201^, ^202^, ^203^ As with other T lymphocyte checkpoints, in vivo blockade of TIGIT in murine tumor models has been shown to reduce Treg frequency, enhance CD8^+^ T cell effector function, and improve antitumor immunity.^200^, ^203^ Additional murine studies reporting co‐expression of TIGIT with other inhibitory checkpoint receptors on T cells have also shown augmented antitumor immunity following combination checkpoint blockade.^195^, ^204^ The potential clinical relevance of this work is highlighted by evidence that combinatorial blockade of TIGIT and other checkpoint receptors also enhances the effector function of ex vivo‐stimulated CD8^+^ TIL from melanoma patients as well as CD4^+^ and CD8^+^ T cells from acute lymphocytic leukemia patients, suggesting that such regimens are likely to show improvements over monotherapies in cancer patients as well.^201^, ^205^ Additionally, alternative approaches for interfering with TIGIT‐mediated suppression of T cells are also emerging. One recent study found that co‐administration of a PD‐1 blocking antibody and an agonistic anti‐glucocorticoid‐induced tumor necrosis factor receptor‐related protein (GITR) antibody improved effector function in CD8^+^ TIL by restoring proper balance to the DNAM‐1/TIGIT signaling axis. Specifically, PD‐1 blockade enhanced DNAM‐1 activity by preventing SHP‐2‐mediated dephosphorylation of its cytoplasmic tail, and GITR agonism reduced TIGIT expression on these cells.^206^ A costimulatory switch receptor that exploits TIGIT to the immune system's advantage was also recently investigated for efficacy in the context of ACT therapy against a xenograft model of established human melanoma. Generated by fusion of the TIGIT exodomain to the immunostimulatory signaling domain of CD28, this costimulatory switch receptor conferred better antitumor immune control by adoptively transferred T cells that had also been transduced to express a MART‐1 Ag‐specific TCR.^207^ Together, these studies highlight multiple strategies for improving antitumor immunity through manipulation of the TIGIT checkpoint in T lymphocytes. With data emerging that CD96 blockade also supports antitumor T lymphocyte function,^193^ it is likely that several members of the PVR family will become important targets for therapeutic intervention in the near future.

### 5.6 Other emerging T lymphocyte checkpoints

Following the successful preclinical and clinical outcomes of ICB regimens targeting the well‐characterized T lymphocyte checkpoints described above, efforts have been under way to identify and elucidate the functional roles of other regulators of T cell activation that might also be targeted in the context of cancer. A number of additional negative regulators have indeed been found to control T lymphocyte function, and insight into these checkpoint pathways has revealed new potential targets for therapeutic intervention. For instance, though it is most highly expressed on DC and other myeloid cell populations, VISTA is also expressed on T lymphocytes and can suppress proliferation and effector cytokine production by both CD4^+^ and CD8^+^ T cells.^208^ Unlike many other checkpoint molecules that are expressed following T cell activation, VISTA is also expressed on naïve T lymphocytes, where it maintains T cell quiescence and immune tolerance and also drives the differentiation of CD4^+^ T cells into FOXP3‐expressing Tregs.^208^, ^209^ Importantly, multiple studies have now highlighted a role for VISTA as a negative regulator of T cell responses to cancer. In a murine glioma model, VISTA‐deficient hosts exhibited enhanced control of implanted tumors that was dependent on CD4^+^ T cells,^210^ and antibody blockade of VISTA in mice bearing transplanted melanomas reduced induction of Tregs within tumors, enhanced CD8^+^ TIL effector function, and delayed tumor progression.^72^ This latter study also reported similar results in an inducible melanoma model and in the MB49 bladder tumor model.

BTLA is another inhibitory checkpoint receptor shared by both DC and T lymphocytes that can contribute to the dysfunction of tumor‐specific T cell responses. In melanoma patients, BTLA is expressed at high levels on Ag‐specific CD8^+^ T cells, and its ligand, HVEM, is expressed on melanoma cells in situ. The potential clinical significance of these findings is underscored by in vitro observations that HVEM‐expressing melanoma cell lines inhibit IFN‐γ production by Melan‐A^MART‐1^ Ag‐specific CD8^+^ T cell clones in a BTLA‐dependent manner.^211^ Indeed, in patients with diffuse large B cell lymphoma, BTLA^+^ T cells from the tumor microenvironment exhibit less cytolytic activity than their BTLA‐deficient counterparts.^212^ Additional evidence for BTLA‐mediated suppression of antitumor T cells comes from a study reporting that BTLA blockade enhances the proliferation, effector cytokine production, and degranulating activity of minor histocompatibility Ag‐specific CD8^+^ T cells isolated from PBMC of patients with hematological malignancies that were treated by allogeneic stem cell transplantation.^213^ BTLA blockade also bolsters the stimulatory effects of PD‐1 blockade on alloreactive CD8^+^ T cells,^214^ suggesting that combinatorial interference with BTLA and PD‐1 could be a useful approach for augmenting antitumor T cell responses in cancer patients. Moreover, in addition to signaling through BTLA, HVEM also binds to the co‐inhibitory receptor CD160, which drives CD8^+^ T cell dysfunction in the context of certain viral infections.^215^ As the frequency of CD160‐expressing CD8^+^ T cells in PBMC populations is higher in esophageal cancer patients than in normal donors^216^ and CD160 expression levels are increased on CD8^+^ T cells from bone marrow of multiple myeloma versus healthy patients,^217^ this checkpoint receptor might also be a useful target for ICB therapy against certain cancer types.

2B4 (CD244) is a member of the signaling lymphocyte activation molecule (SLAM) family of immunoreceptors. Though activation signals can indeed be transmitted through 2B4 via its CD48 ligand, the ultimate outcome of receptor engagement appears to be controlled by the ratio of 2B4 to SLAM adaptor protein (SAP) content in a given cell. According to the current model, under conditions where 2B4 expression is limited, the intracellular adaptor protein SAP can bind to 2B4's cytoplasmic ITSM signaling motifs and prevent the binding of inhibitory phosphatases, thus enabling delivery of activation signals. On the other hand, elevated expression of 2B4 on exhausted T cells, as has been reported in many cancers,^217^, ^218^, ^219^ shifts the 2B4:SAP balance such that intracellular SAP levels are insufficient to prevent phosphatase‐mediated inhibitory signaling.^220^ Although the antitumor activity of 2B4 blockade has yet to be investigated, blockade of this checkpoint receptor does improve overall T cell effector function and survival in an animal model of sepsis with preexisting malignancy,^221^ and blockade of either CD48 or 2B4 reverses CD8^+^ T cell exhaustion in the context of chronic viral infection.^222^, ^223^ Therefore, as the regulation of antitumor T cell responses by the CD48/2B4 axis continues to be investigated, checkpoint inhibitors targeting this pathway may soon be added to the repertoire of ICB agents available for cancer therapy.

NKG2A, an inhibitory checkpoint classically associated with natural killer (NK) cells, has also been found to negatively regulate T lymphocytes. The receptor for the nonclassical MHC class I molecules Qa‐1 in mice and HLA‐E in humans, NKG2A mediates inhibitory signaling via its cytoplasmic ITIM domains. In murine models, two groups have recently shown that NKG2A blockade enhances CD8^+^ T cell‐dependent control of tumors, both in the context of naturally occurring and vaccine‐induced immune responses.^224^, ^225^ NKG2A expression on TIL was also recently found to be a poor prognostic factor for overall survival of colorectal cancer patients.^226^ An NKG2A blocking antibody, monolizumab, is currently under investigation as an ICB therapeutic, and an interim report of a Phase II trial has shown clinical benefit of monolizumab in combination with cetuximab for SCCHN patients.^227^

**FIGURE 3:**
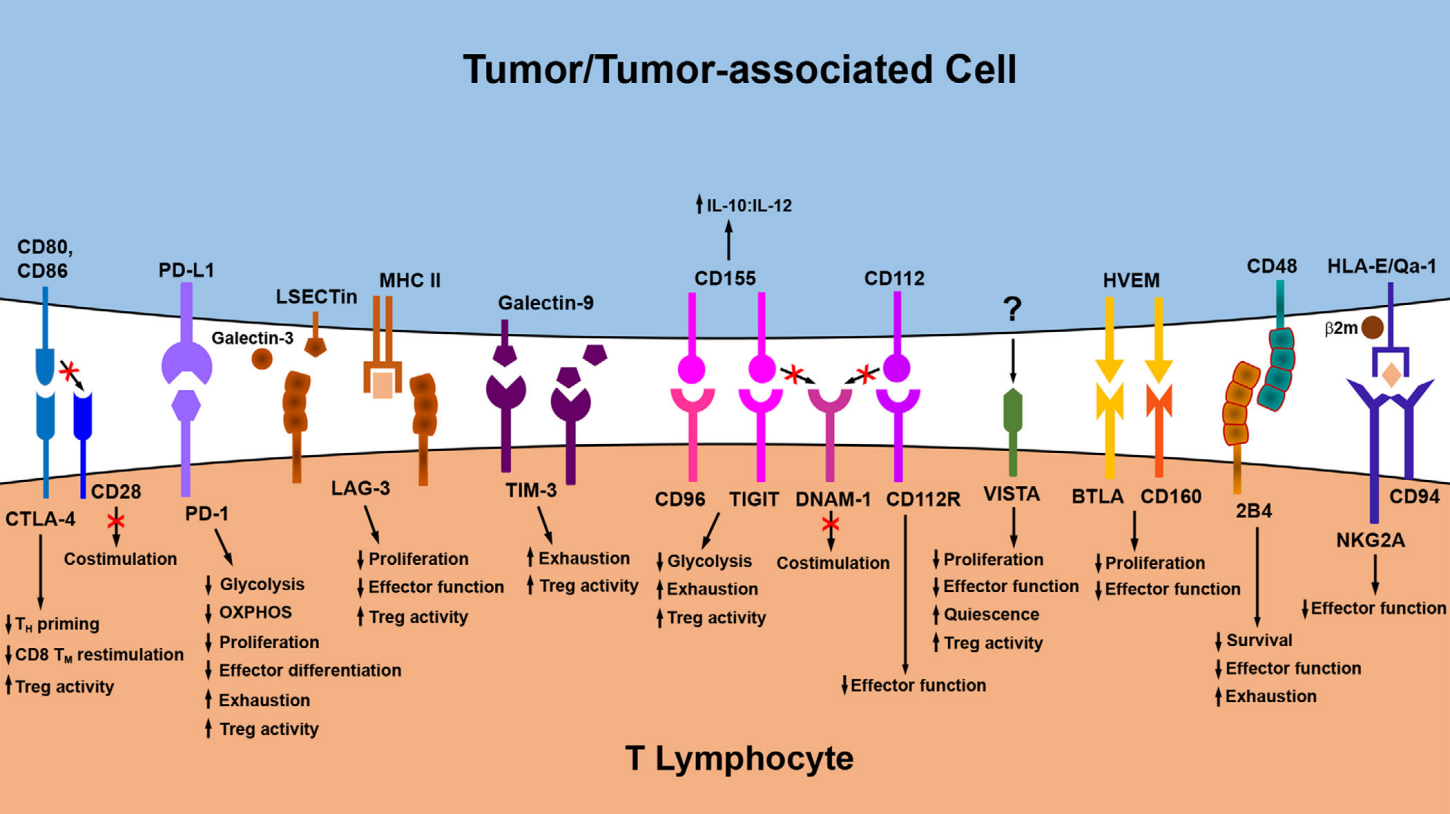
Immunologic checkpoints influencing T lymphocyte function in the TME. Several inhibitory checkpoint pathways that negatively regulate antitumor T lymphocyte function have now been identified. Engagement of these checkpoint receptors by ligands expressed on/by tumor cells and other populations within the TME may compromise antitumor immunity by interfering with the expansion, effecter differentiation, and survival of tumor‐specific T cells. Checkpoint inhibitors and other therapeutic strategies that disrupt engagement of these pathways in T lymphocytes can improve antitumor immunity by negating the signals delivered through these inhibitory receptors, as discussed in more detail in the main text. Abbreviations in this figure not defined in the main body of the text: T_M_, memory T cell

With the discovery of such a vast array of inhibitory checkpoints for T lymphocytes over the last two decades, there now exists a multitude of realized and potential therapeutic targets for promoting/restoring T cell reactivity against cancer (Figure 3). As these and newly discovered checkpoint pathways continue to be investigated, the challenge going forward will lie in identifying the particular checkpoints that should be targeted by combinatorial or sequential ICB therapies in specific cancer patients. To this end, tumor immune profiling,^228^ not only prior to treatment but also in cases of disease relapse, will become critical to the success of ICB‐based therapies, as it will reveal the specific cohort of immune checkpoint receptors and ligands expressed by an individual's T lymphocytes and other cell populations in the TME. Based on the successes already seen with initial approaches to combination ICB therapy, it is very likely that incorporating additional inhibitors of targetable checkpoints into ICB regimens will further improve the efficacy and durability of antitumor immune responses in many cancer patients.

## 6 METABOLIC CHECKPOINTS INFLUENCING T CELL FUNCTION IN THE TUMOR MICROENVIRONMENT

**FIGURE 4:**
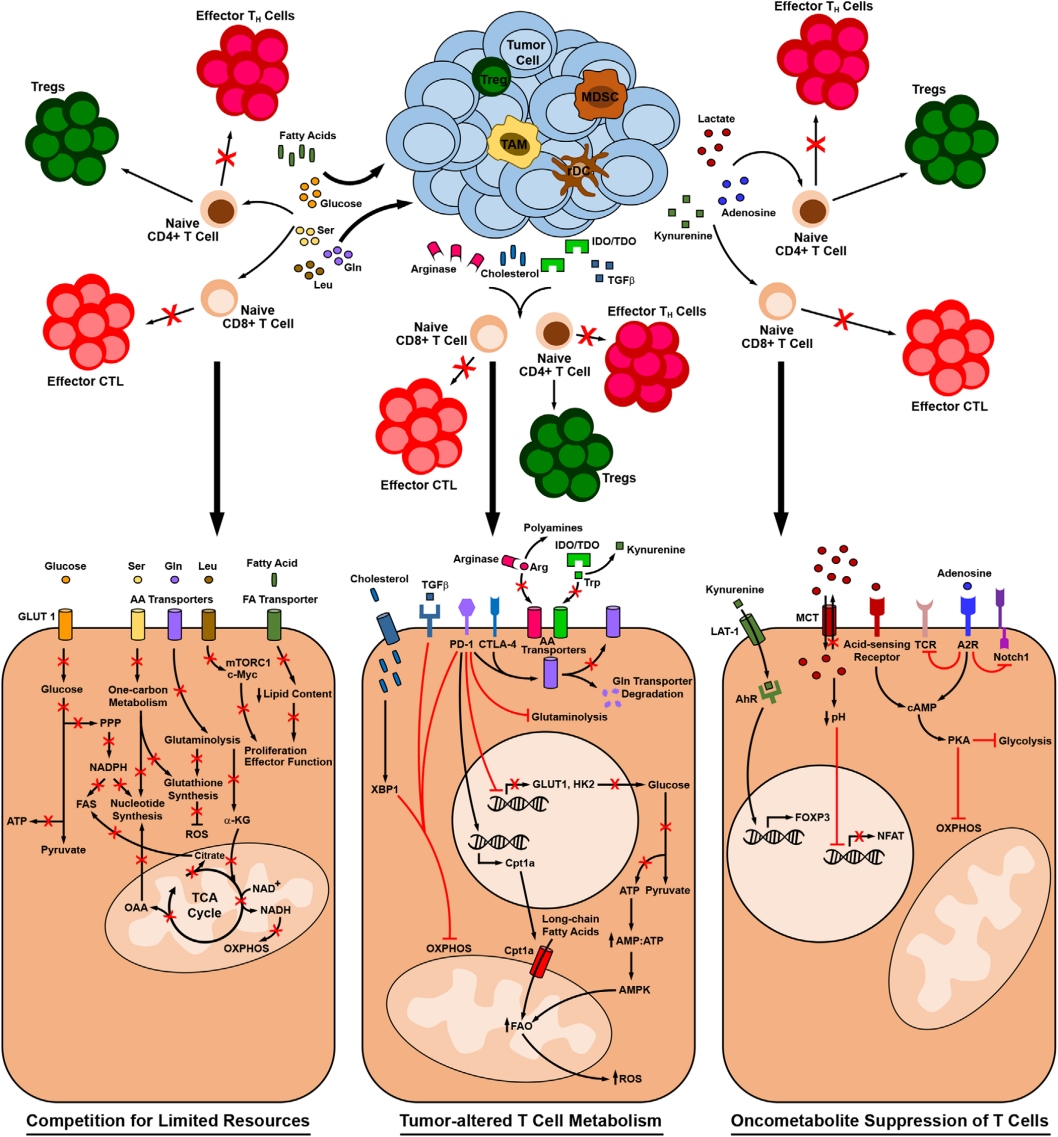
Metabolic regulation of T lymphocyte function within the TME. T lymphocyte metabolism is tightly linked with immune function and may be dysregulated within the TME in several ways. Competition between T lymphocytes and tumor cells for limiting resources can result in a lack of nutrient support for metabolic pathways essential to T cell activation and effector differentiation. Immunosuppressive cytokines, enzymes, and cholesterol derived from tumors and tumor‐associated cells can all impede metabolic pathways that support antitumor T cell function while also driving alternative pathways associated with immune tolerance. Finally, accumulation of suppressive oncometabolites produced by metabolically active tumors and tumor‐associated cells suppress metabolic pathways essential to T cell activation and drive the differentiation of tumor‐supporting Tregs. As described in more detail in the main text, insight into the metabolic regulation of T lymphocyte function within the TME has yielded several targets for therapeutic strategies aiming to improve the metabolic fitness and immune function of antitumor T cells. Note that the magnified intracellular signaling and metabolic pathways shown reflect events that have been reported in either CD4^+^ or CD8^+^ T cells. Abbreviations in this figure not defined in the main body of the text or in other figure legends: rDC , regulatory DC; TAM, tumor‐associated macrophage; FA, fatty acid; AA, amino acid, α‐KG, α‐ketoglutarate; OAA, oxaloacetate; HK2, hexokinase 2; AhR, aryl hydrocarbon receptor

Just as DC undergo metabolic reprogramming to support the acquisition of immune‐stimulating functions during their maturation and activation, T lymphocytes also modulate their metabolism following activation so that they may fulfill the biosynthetic and bioenergetic requirements of clonal expansion, effector differentiation, and memory formation. Shifts in carbohydrate, lipid, and amino acid metabolism all contribute to the progression of a T cell through its various stages of an immune response, and interference with any of these metabolic pathways can therefore have negative consequences on the immunoreactivity of these cells. In this regard, similar to its impact on DC metabolism, the TME also poses metabolic constraints on T lymphocytes, with competition for limited resources, regulation of T cell metabolism, and accumulation of immunosuppressive metabolic by‐products all acting as significant barriers to antitumor T cell function (Figure 4). Nevertheless, with recent insights into the metabolic dysfunction of T lymphocytes in the context of cancer, therapeutic strategies that restore immune‐supporting metabolic pathways in T cells and that overcome the deleterious effects of immunosuppressive metabolites on T cells within the TME are now emerging as viable options for improving the quality of antitumor T cell immunity.

### 6.1 Tumor‐imposed limitations on T lymphocyte carbohydrate/energy metabolism

Though naïve T lymphocytes are relatively inactive in terms of their metabolism, achieving their modest energy needs with low rates of OXPHOS fueled by intermediates derived from glucose, glutamine, and fatty acid metabolism,^229^ T cells undergoing activation and effector differentiation are metabolically reprogrammed toward aerobic glycolysis, utilizing glucose as a substrate for various biosynthetic pathways more so than as a source for TCA‐driven OXPHOS.^230^ In particular, recently activated T lymphocytes upregulate expression of both GLUT1 and LDHA, which enhance glucose uptake and divert pyruvate away from the TCA cycle, respectively.^231^, ^232^ The resulting commitment to glycolytic metabolism enables ATP production while also allowing intermediates from this pathway to be directed to the PPP for increased nucleotide biosynthesis and NADPH‐driven FAS, both of which are necessary to support rapid cell division during clonal expansion. In addition to meeting these biosynthetic requirements for T cell proliferation, glycolytic metabolism during T cell differentiation is also essential for acquisition of effector function. In this regard, Peng *et al* found that LDHA upregulation and reduced TCA cycle activity in stimulated T lymphocytes contribute to maintenance of high levels of acetyl‐coenzyme A, a substrate for histone acetylation needed to activate *Ifng* gene expression.^232^ More recently, activation of glycolysis was also found to occupy LDHA and inhibit its binding to the 3′‐UTR of mRNAs encoding IFN‐γ, TNF‐α, and IL‐2, thereby preventing an interaction that otherwise represses translation of these cytokines.^233^ A similar mechanism has also been reported by Chang *et al*, who found that glycolysis supports IFN‐γ‐based effector activity in T lymphocytes by maintaining glucose‐driven engagement of the glycolytic enzyme GAPDH and in turn preventing its own translational repression of IFN‐γ mRNA.^234^ Importantly, in addition to enhancing effector cytokine production, glycolytic metabolism also supports expression of genes encoding the perforin and granzyme molecules essential for cytolytic effector function.^235^

It has also become apparent that increased glycolytic metabolism during T cell activation does not come at the expense of OXPHOS, which is also upregulated in these cells in order to meet the bioenergetic demands that accompany their activation. In addition to supporting ATP production in activated T cells, mitochondrial metabolism also yields ROS important for activating specific cell signaling pathways that lead to clonal expansion.^236^ With glucose serving as the substrate for aerobic glycolysis in activated T lymphocytes, OXPHOS is instead driven primarily by metabolism of glutamine in these cells,^230^, ^237^, ^238^ as discussed in more detail below. Finally, though the transition from effector to memory T cells is associated with a shift from aerobic glycolysis to FAO‐based metabolism, glucose is still necessary to support the *de novo* synthesis of lipids for subsequent oxidation.^239^, ^240^ In memory cells, FAO enhances spare respiratory capacity, supporting mitochondrial function that not only promotes the long‐term survival of these cells but that is also key to the rapid induction and maintenance of both aerobic glycolysis and OXPHOS, which are again upregulated during recall responses.^241^, ^242^

Because glucose is such an essential nutrient for T cell activation and differentiation, competition for this resource between T lymphocytes and glycolytically active tumor cells poses significant limitations on the efficacy of antitumor T cell responses. To this point, experimental work in a progressor versus regressor tumor model has highlighted the relevance of this competition to antitumor T cell function. In in vitro studies, activated T lymphocytes co‐cultured with highly glycolytic progressor tumor cells exhibited reduced effector activity as compared to T cells co‐cultured with regressor tumor cells that consume less glucose, and glucose supplementation was sufficient to restore the effector function of T cells in progressor tumor co‐cultures.^82^ Similarly, in vivo studies in this model demonstrated that glucose levels were reduced in the TME of progressor versus regressor tumors, and TIL isolated from progressor tumors displayed a lower extracellular acidification rate (indicative of reduced glycolytic metabolism) and produced less IFN‐γ than TIL from regressor tumors. Selection of glycolytically active cells from regressor tumor populations or retroviral transduction of regressor tumor cells with glycolysis‐promoting genes were each also sufficient to confer escape from T lymphocyte‐mediated control of tumor outgrowth. Interestingly, this and several other studies have also documented a link between various immunologic checkpoint pathways and glycolytic dysfunction in T cells,^82^, ^147^, ^148^, ^196^ demonstrating that competition for glucose is not the only mechanism by which glycolysis can be compromised in T cells within the TME. Indeed, glycolysis can be inhibited in T lymphocytes in a variety of ways. For instance, PD‐1 signaling in T cells downregulates expression of the GLUT1 and hexokinase 2 proteins needed to import and subsequently initiate breakdown of glucose.^147^ On the other hand, in CD8^+^ TIL from renal cell carcinoma patients, reduced glucose uptake was associated not with reduced expression of GLUT1 but instead with a loss of phosphorylation of this transporter, a phenomenon that authors attributed to a probable failure of dephosphorylated GLUT1 to traffic to the cell surface.^243^ This same study also reported downregulation of the glycolytic enzyme GAPDH in CD8^+^ TIL, highlighting another potential means of compromising this pathway downstream of glucose uptake. Another glycolysis‐regulating enzyme, enolase 1, is dysregulated by multiple checkpoint pathways, and reduced glycolytic metabolism of CD8^+^ TIL from B16 melanomas was associated with a loss of enolase 1 enzymatic activity when compared to acute T effector cells.^244^ Interestingly, enolase 1 catalyzes formation of phosphoenolpyruvate, a glycolytic metabolite that sustains TCR‐mediated Ca^++^/NFAT signaling and effector function in both CD4^+^ and CD8^+^ T cells,^245^ highlighting the significance of glycolysis not only as a means of achieving bioenergetic and biosynthetic needs in activated T lymphocytes but also as a mediator of stimulatory signaling in these cells.

Mitochondrial dysfunction has also been reported in TIL isolated from multiple cancer types. In addition to the aforementioned glycolytic defects in CD8^+^ TIL from renal cell carcinoma patients, mitochondria in these cells also exhibited morphological abnormalities (small and fragmented) and were hyperpolarized, producing elevated levels of ROS that limited TIL effector function.^243^ Mitochondrial mass and function were also found to be reduced in CD8^+^ TIL isolated from murine melanomas, colon adenocarcinomas, and lung carcinomas, as well as from patients with HNSCC.^246^ These defects were associated with reduced T lymphocyte expression of PGC1α, a protein critical for mitochondrial biogenesis. Others have shown that tumor‐derived TGF‐β impairs mitochondrial respiration in T cells^247^ and that PD‐1 signaling alters mitochondrial structure and limits this organelle's capacity for OXPHOS.^248^

Based on the glycolytic and mitochondrial deficiencies observed to date in tumor‐associated T lymphocytes, there has been great interest in exploring strategies to metabolically reprogram these cells in order to improve their function within the TME. T cell activation markers and cytokine secretion have been at least partially restored by metabolic reprogramming of TIL ex vivo, either by provision of pyruvate or phosphoenolpyruvate to bypass glycolytic defects or by treatment with mitochondrial ROS scavengers,^243^, ^244^ suggesting that interventions targeting these metabolic abnormalities might indeed have therapeutic benefit. To this point, overexpressing the PCK1 enzyme that converts oxaloacetate into phosphoenolpyruvate in adoptively transferred tumor‐specific CD4^+^ or CD8^+^ T cells improved the control of established B16 melanomas.^245^ Likewise, overexpression of PGC1α in tumor‐specific CD8^+^ T cells enhanced mitochondrial function, effector activity, and the ability of adoptively transferred cells to control established tumors.^246^ Together, these studies highlight the significance of biosynthetic and bioenergetic pathways as regulators of T cell function in the TME, and they offer great promise for metabolic manipulation of T lymphocytes as a means of enhancing the antitumor immune reactivity of these cells.

### 6.2 Lipid metabolism as a regulator of T cell function within the TME

As described above, the metabolic shift toward aerobic glycolysis during T cell activation is accompanied by a shift away from FAO as a major driver of OXPHOS. Rather than catabolizing lipids through lipolysis and subsequent FAO, these activated T cells instead switch to FAS in order to increase cell and organelle membrane biomass as they undergo clonal expansion. Increased fatty acid content is also likely to support effector differentiation of these cells as well, as lipid‐dependent posttranslational modifications have been found to enhance the activity of signaling pathways associated with this process.^249^, ^250^ In recently activated T cells, the increase in lipid content is driven not only by de novo lipogenesis but also by the uptake of exogenous fatty acids.^230^, ^240^, ^251^, ^252^ Likewise, tissue‐resident memory T cells also take up exogenous fatty acids to support their long‐term survival.^253^ At the same time, it is well‐established that tumor cells upregulate expression of fatty acid transporters and scavenger receptors such as CD36 so that they too may increase uptake of exogenous fatty acids and lipoproteins to support cell growth, survival, energy production, and a variety of oncogenic functions associated with tumor progression.^254^, ^255^, ^256^, ^257^, ^258^, ^259^, ^260^, ^261^ Therefore, competition between T lymphocytes and tumor cells for lipids within the TME may limit fatty acid acquisition by T cells, thus compromising the expansion and differentiation of recently activated cells as well as the long‐term persistence of more fully differentiated cells.

While tumor cells may deplete the TME of particular lipids important for T lymphocyte function, they may also promote accumulation of other lipids that suppress T cell responses. For instance, in several murine tumor models, it has been reported that cholesterol levels are enriched in tumor tissue as compared to normal tissues and that the cholesterol content of exhausted CD8^+^ TIL isolated from both murine tumors and cancer patients is elevated compared to that in T cells isolated from non‐cancerous tissue.^262^ Cholesterol accumulation in TIL drives exhaustion by promoting expression of the ER stress sensor XBP1, a transcription factor that directly upregulates expression of PD‐1 and 2B4. Importantly, this and a related study highlighted several interventions that could improve antitumor immune function by interfering with cholesterol metabolism or its downstream effects, including treatment of tumor‐bearing mice with an ER stress inhibitor, adoptive transfer of XBP1‐knockdown tumor‐specific CD8^+^ T cells, treatment of CD8^+^ T cells with β‐cyclodextrin to deplete cholesterol prior to adoptive transfer, and pharmacologic reduction of cholesterol content in the TME.^262^, ^263^ Another group has also shown that cholesterol esterification, rather than cholesterol uptake or biosynthesis, is the primary factor influencing T lymphocyte dysfunction, and treatment of tumor‐bearing mice with an inhibitor of the ACAT1 enzyme that promotes cholesterol esterification increased the number of effector and effector memory CD8^+^ TIL and resulted in delayed tumor progression.^264^

In addition to regulating lipid content within the TME, tumor cells can also exert more direct influence over lipid metabolism by T cells. Glucose depletion by tumor cells can yield higher AMP:ATP ratios within T lymphocytes and therefore activate signaling via the nutrient sensor AMPK, a known driver of FAO.^265^ While such metabolic plasticity may enable T lymphocytes to generate ATP and survive in glucose‐depleted environments, FAO does not support the effector differentiation of these cells necessary for antitumor reactivity. Even in a TME where glucose is not limiting, the engagement of multiple checkpoint pathways can also suppress glycolytic metabolism in T lymphocytes, thus requiring these cells to adopt alternative metabolic pathways, including FAO, to meet their bioenergetic demands. Indeed, PD‐1 signaling impedes effector differentiation in part by upregulating expression of CPT1 mitochondrial fatty acid transporters and directly reprogramming T cells toward FAO.^147^, ^248^, ^266^ Of significance, while such reprogramming is detrimental to the differentiation of CD8^+^ T cells and the T_H_1, T_H_2, and T_H_17 subsets of CD4^+^ T cells, FAO actually favors the induction of Tregs, which may utilize glycolysis but rely on it less heavily for their differentiation.^267^, ^268^ Interestingly, lipid availability to Tregs was recently shown to control mitochondrial integrity and suppressive activity, with insufficient lipid uptake driving increased IL‐10 production via mtDNA release from damaged mitochondria and subsequent activation of the cGAS‐STING‐type 1 IFN pathway.^269^ Based on these findings, it is interesting to speculate that tumors may not only foster the “development” of Tregs by promoting FAO in these cells at early stages of tumor progression but that they may also drive the “suppressive activity” of these cells as they continue to proliferate and deplete lipids from the TME over time.

The shift from FAO to glycolytic metabolism is clearly important during early stages of T lymphocyte activation and is therefore significant to the induction of natural antitumor T cell responses. Likewise, the antitumor efficacy of ex vivo‐generated CTL used for ACT therapy is also dependent on glycolysis and can therefore be restricted by the glycolytic activity of the tumors they target.^270^ As discussed above, metabolic interventions that support glycolytic metabolism by T cells are therefore attractive strategies for promoting T cell effector function within the hostile TME. Alternatively, while FAO fails to support potent T cell effector differentiation in vivo, insights into the role of this pathway in promoting T lymphocyte survival and memory development have spearheaded efforts to manipulate FAO as a means of generating long‐lived cells for ACT‐ and CAR‐T‐based cancer therapies. In this regard, Sukumar *et al* found that inhibiting glycolysis during ex vivo expansion of Ag‐specific T cells resulted in acquisition of a memory phenotype and led to greater accumulation of these cells in tumors following adoptive transfer.^271^ In keeping with the ability of FAO to support memory T cell engagement of glycolysis and OXPHOS during recall responses,^242^ when these adoptively transferred cells were re‐isolated from B16 melanomas 5 days post‐transfer, they expressed high levels of glycolytic enzymes and displayed augmented effector functions, consistent with their improved ability to control tumor outgrowth. Similar data in the B16 melanoma model was reported by Crompton *et al*, who also showed that human TIL expanded under glycolysis‐inhibiting conditions exhibited greater survival following transfer into a humanized mouse model, suggesting this approach might also improve the antitumor efficacy of ACT therapy in cancer patients.^272^ In contrast to these studies that have employed inhibitors of glycolysis to modulate T cell metabolism during ex vivo production of T lymphocytes for ACT therapy, others have shown more direct evidence that maneuvers to enhance FAO improve the persistence and antitumor reactivity of adoptively transferred T cells.^273^, ^274^ It has also been shown that the design of CAR‐T cells influences metabolic activity and that FAO‐promoting CARs enhance survival of these cells in vitro. Specifically, chimeric TCRs fused to 4‐1BB signaling domains are stimulated to undergo FAO and survive longer than those with TCRs fused to CD28 signaling domains, which instead preferentially undergo glycolysis upon stimulation.^275^ While this study did not examine T cell survival or antitumor activity in vivo, these data suggest that conditions favoring FAO metabolism in CAR‐T cells might indeed translate to improved therapeutic efficacy post‐adoptive transfer. Future studies will indeed be necessary to confirm this hypothesis and to identify the optimal conditions for generating robust CAR‐T cells, but at this point it is clear that metabolic considerations will play a key role in the design of these as well as non‐chimeric ACT‐based cancer therapies going forward.

### 6.3 Deficiencies in T lymphocyte amino acid metabolism in the TME

As interest in T cell and cancer metabolism has increased in recent years, a number of amino acids have emerged as critical regulators of the outcome of T lymphocyte/tumor cell interactions within the TME. Along with the metabolic switch to aerobic glycolysis during early stages of T lymphocyte activation, T cells also increase their metabolism of glutamine, upregulating expression of membrane transport proteins that promote glutamine uptake and increasing the activity of enzymes that drive subsequent glutaminolysis.^237^ Like glycolysis, glutaminolysis also supports biosynthetic pathways in activated T cells and is critical to both proliferation and cytokine production by these cells. Additionally, glutamine metabolism plays an equally important role in the generation of α‐ketoglutarate, a TCA cycle intermediate that supports additional ATP production through oxidative metabolism^230^, ^237^ and that drives effector, rather than Treg, differentiation.^238^, ^276^ Glutaminolysis further supports effector T cell differentiation and survival by driving glutathione synthesis and in turn allowing T lymphocytes to maintain redox homeostasis.^277^, ^278^ That glutaminolysis is enhanced by AMPK signaling during glucose deprivation highlights in particular the utility of this pathway as a means of maintaining T cell biosynthetic and bioenergetic programs in a glucose‐limiting environment,^279^ such as that often encountered in the TME. However, the same benefits that glutamine provides to T lymphocytes also exist for cancer cells themselves, which likewise increase glutamine uptake and metabolism as part of their own metabolic reprogramming and therefore compete with T cells for use of this resource.^280^ In addition to this competition, engagement of T lymphocyte checkpoints can also directly interfere with glutamine metabolism in much the same way as described above for other metabolic pathways. Both PD‐1 and CTLA‐4 signaling inhibit expression of glutamine transporters on T cells, and PD‐1 signaling also suppresses glutaminolysis within these cells.^147^

Because of its role in supporting cancer progression, there has been interest in targeting glutamine metabolism in cancer cells therapeutically. While the evidence that T cells rely on glutamine uptake and metabolism during their activation suggests that such interventions might also compromise the metabolic fitness of T cells within the TME, recent studies have revealed that at least in certain contexts the metabolic plasticity of T cells may be greater than that of cancer cells, thereby enabling survival and maintenance of effector activity in the face of glutaminolytic inhibition. Though chronic or complete inhibition of glutaminolysis impaired T lymphocyte function in vivo, transient inhibition in vitro enhanced the survival and effector function of adoptively transferred T_H_1 and CD8^+^ T cells,^281^ suggesting that these cells might be able to provide more durable antitumor immune reactivity. Pharmacologic blockade of glutamine metabolism in MC38 tumor‐bearing mice also supported antitumor T cell function, suppressing not only glutaminolysis but also glutamine‐driven glycolytic metabolism in tumor cells. In addition to these direct antitumor effects, inhibiting these pathways in cancer cells also restored nutrient availability within the TME, allowing metabolically flexible T cells to exploit alternative pathways that enhanced their proliferation, survival, and antitumor effector function.^282^

Competition between tumor cells and T lymphocytes for serine and leucine can also compromise the metabolic fitness of T cells within the TME. Acquisition of extracellular serine is necessary to support the proliferation of activated T cells, which rely on this amino acid as a source of one‐carbon metabolism for de novo nucleotide biosynthesis.^283^ However, uptake of exogenous serine is utilized for this same purpose by cancer cells, which otherwise exhibit defects in both proliferation and survival under conditions of serine deprivation.^284^, ^285^ T lymphocytes also upregulate expression of the Slc7a5 leucine transporter following activation, and sustained uptake of leucine is necessary to maintain mTORC1 activation and c‐Myc expression, both of which drive clonal expansion and effector differentiation.^286^ However, as Slc7a5 and another leucine transporter, Slc43a1, are also upregulated on many tumor cell types,^287^, ^288^ leucine uptake by these cells could ultimately disrupt the antitumor functionality of T cells by starving them of this important amino acid within the TME.

In addition to tumor cells, other cell types that frequently accumulate within the TME also interfere with metabolism of amino acids important for T cell function. Myeloid‐derived suppressor cells (MDSC), M2‐like tumor‐associated macrophages, and immunoregulatory DC have all been shown to produce high levels of arginase I within tumor tissue^289^, ^290^, ^291^, ^292^, ^293^ and can therefore deplete the TME of arginine, an amino acid critical to the survival and antitumor reactivity of T lymphocytes.^294^ Arginase activity also promotes the induction of Tregs, further contributing to the immunosuppressive environment within a tumor.^295^ Moreover, though the extent to which arginine is utilized by DC during their activation is currently unknown, it has been reported that polyamine by‐products of arginine metabolism do condition DC to acquire an immunosuppressive phenotype,^296^ and this pathway may also contribute to T cell dysfunction within a TME where arginine is heavily metabolized. Interestingly, this study revealed that arginine‐derived polyamines promoted DC expression of enzymatically active IDO‐1, a potent inducer of tryptophan catabolism. Tryptophan is another amino acid critical to antitumor T cell function, and IDO‐1 expression by tumor‐associated MDSC and regulatory pDC is a frequently reported mechanism of tumor immune escape.^297^, ^298^, ^299^ Another tryptophan‐catabolizing enzyme, tryptophan‐2,3‐dioxygenase (TDO), is also expressed in several human tumor types and has been shown to limit the immunologic control of murine tumors.^300^, ^301^

A number of small molecule inhibitors targeting IDO and TDO have now been developed and are in clinical trials for various cancer types,^302^ and a first‐in‐class arginase inhibitor (CB‐1158) that has been shown to enhance antitumor T cell reactivity in murine models is also now in early phase clinical trials.^303^ Though data has yet to be released for the arginase inhibitor trials, the outcomes of trials for IDO inhibitors administered either as single agents or as part of combinatorial regimens with checkpoint blockade therapy have unfortunately been met with limited success.^304^, ^305^ Off‐target effects and tumor‐acquired resistance have both compromised the efficacy of various IDO inhibitors developed to date and are issues that will need to be addressed in the future. To overcome the issue of off‐target effects, strategies to interfere with IDO‐mediated immune suppression that do not involve small molecule inhibitors have been devised. In this regard, targeted silencing of IDO in endogenous APC improves antitumor T cell function in a murine melanoma model,^306^ and silencing IDO in exogenous DC prior to vaccination improves T cell function and tumor control in a murine breast cancer model.^307^ A case report describing this latter strategy in a melanoma patient has revealed that this approach may indeed show immunologic and antitumor efficacy in humans as well.^308^ In addition, for IDO and TDO inhibitors that do have tolerable safety profiles, it is hoped that the issue of tumor‐acquired resistance may be overcome by combinatorial administration of these inhibitors or the use of dual‐targeting IDO/TDO inhibitors, both of which might prevent tumor escape from more selective drugs that fail to inhibit an alternative tryptophan‐catabolizing pathway when administered as single agents.

### 6.4 Suppression of T lymphocytes by oncometabolites in the TME

The same oncometabolites that compromise DC function within the TME also suppress the function of T lymphocytes. First reported in the context of tumor spheroid and other in vitro settings, the accumulation of extracellular lactic acid was found to disrupt lactate export by glycolytically active T cells and to promote its buildup intracellularly in these cells, leading to suppressed proliferation, cytokine secretion, and cytotoxic function.^309^ Subsequent work has since shown that lactic acid secretion by highly glycolytic tumors also blunts antitumor T cell functions in vivo. LDHA expression and lactic acid production by tumor cells correlates with suppression of TIL effector function, likely as a result of the poor lactate efflux and resulting intracellular acidification that interferes with NFAT expression in T cells.^310^ Evidence has also emerged that T cell function can be compromised by lactate signaling via acid‐sensing receptors.^311^ The significance of lactate accumulation to antitumor T cell dysfunction is highlighted by studies exploring approaches to interfere with acidification in the TME. Proton pump inhibitors, nonsteroidal anti‐inflammatory drugs that block lactate secretion by tumors, and bicarbonate therapy have all been shown to neutralize TME acidity and enhance either natural or therapy‐associated antitumor T cell responses.^311^, ^312^, ^313^

As discussed above, lactic acid also indirectly influences T cell function within the TME by reprogramming intratumoral pDC to support Treg induction via increased tryptophan metabolism.^101^ In addition to depleting this essential amino acid necessary for antitumor T cell function, another consequence of IDO/TDO activity in the TME is the release of kynurenine as a by‐product of tryptophan metabolism. Kynurenine interacts with the aryl hydrocarbon receptor on CD4^+^ T cells and promotes expression of FOXP3,^314^ which in turn programs Treg metabolism to support survival and maintenance of immune regulatory function in low‐glucose, high‐lactate environments.^315^ In this way, oncometabolites derived from both carbohydrate and amino acid metabolism act in concert to create an effector T cell/Treg imbalance that ultimately fosters immune escape and tumor progression.

Adenosine is another oncometabolite enriched in the TME that represses T lymphocyte function and promotes Treg accumulation, primarily via the A_2A_ receptor. Engagement of this receptor promotes intracellular accumulation of cAMP and interferes with membrane proximal TCR signaling and activation of the Notch1 pathway, leading to reduced proliferation, cytokine secretion, and cytotoxic activity.^316^, ^317^, ^318^ Underscoring the role of metabolism in the regulation of these various T cell functions, it was recently shown that adenosine‐driven cAMP accumulation also activates PKA signaling, which in turn suppresses mTORC1‐driven glycolysis and OXPHOS.^319^ Together, these studies offer mechanistic explanations for the well‐documented link between adenosine in the TME and dysfunctional TIL.^320^, ^321^, ^322^ Based on these and other reports, a number of strategies for interfering with adenosine‐mediated suppression of antitumor T cells have been investigated, including some that have made their way into clinical trials. Pharmacologic blockade of the A_2A_ receptor reduces the frequency of peripheral and intratumoral Tregs and enhances not only tumor infiltration by CD8^+^ T cells but also the effector function of these cells.^323^, ^324^ Likewise, genetic ablation of the A_2A_ receptor enhances the antitumor efficacy of both traditional and CAR‐T‐based ACT therapies.^322^, ^325^ Consistent with the role of tumor hypoxia in the generation of extracellular adenosine and upregulation of the A_2A_ receptor,^326^ hyperoxic therapy can also reverse hypoxia‐adenosinergic immunosuppression within the TME, leading to improved antitumor reactivity by both endogenous and adoptively transferred CD8^+^ T cells.^327^ Finally, blockade of CD39 and CD73, as well as another adenosine‐generating ectoenzyme belonging to the ADP ribosyl cyclase family, CD38, improves the functional quality of antitumor T cells.^328^, ^329^ These latter approaches in particular may support antitumor T cell function in multiple ways, as evidenced by a recent study demonstrating that CD39 blockade not only reduces the level of adenosine available for direct suppression of T cells but also promotes immunogenic signaling by extracellular ATP through P2X7 purinergic receptors on myeloid cell populations.^330^ This finding may also explain the synergistic effects of CD39 blockade in combination with oxaliplatin chemotherapy,^329^ which itself promotes ATP release from apoptotic tumor cells and may therefore support purinergic receptor signaling under conditions in which released ATP is not quickly converted to adenosine.

Collectively, the studies described herein reveal great promise for metabolic interventions as a strategy to support the induction and maintenance of antitumor immune responses. They also highlight the significance of tumor metabolomic profiling as a useful tool for designing appropriate treatment plans in cancer patients. As such profiling continues to become more commonplace in clinical settings,^331^, ^332^, ^333^, ^334^ our understanding of how particular metabolic signatures are likely to influence antitumor immune responses will have increasing prognostic value in predicting patient response to immunotherapy, and it will therefore allow for more effective patient stratification toward particular treatment regimens most likely to achieve clinical benefit. As discussed in more detail below, these metabolomic insights are also capable of informing the design of combinatorial regimens that may ultimately increase the frequency of patients who benefit from immunotherapy, as such regimens can ultimately yield a TME that is more metabolically permissive to antitumor immune reactivity.

## 7 COMBINATORIAL APPROACHES TO CANCER THERAPY: TAKING AN OLD IDEA TO NEW HEIGHTS

There is nothing new about the idea of combinatorial therapy for cancer. Indeed, the history of oncology is replete with examples of combinatorial approaches designed to attack cancer from multiple angles. However, a recurring theme over the years has been that while such approaches have achieved success in some cases, their initial promise has never been fully realized, typically due to a poor understanding of cancer biology. Now more than ever, with advances in our understanding of the basic biology of both the immune system and the cancer cell, the field of oncology seems truly poised for significant breakthroughs in both targeted and immunotherapeutic approaches to treatment in the years ahead, and the potential for achieving durable clinical benefits from combinatorial regimens that support the antitumor functions of both DC and T cells is perhaps the most promising for cancer therapy to date.

Based on their success in the clinic, a number of checkpoint inhibitors have received FDA approval for use as cancer therapeutics (Table 1). In particular, recent trials of combinatorial ICB therapy have been extremely promising, leading to unprecedented outcomes in the clinical management of multiple cancer types. Phase III clinical trials for ipilimumab + nivolumab combination therapy have produced staggering overall survival statistics, with a 5‐year survival rate of 52% for advanced melanoma patients^154^ and a 2‐year survival rate of 40% for stage IV or recurrent NSCLC patients whose tumors are characterized by PD‐L1 expression levels ≥1%.^155^ With the discovery of additional immunologic checkpoints beyond CTLA‐4 and PD‐1/PD‐L1 and ongoing development of novel checkpoint inhibitors targeting these pathways in both DC and T lymphocytes (Figures 1 and 3), it is reasonable to expect in the near future that new combinations will further increase either the percentage of patients responding to ICB therapy or the duration of responses elicited in the subset of patients who do benefit from these regimens.

**TABLE 1:** Summary of FDA‐approved immunologic and metabolic checkpoint inhibitors

Target	Therapeutic	Class of Drug
CTLA‐4	Ipilimumab (Yervoy®)	Humanized IgG1 mAb
PD‐1	Nivolumab (Opdivo®)	Fully human IgG4 mAb
	Pembrolizumab (Keytruda®)	Humanized IgG4 mAb
	Cemiplimab (Libtayo®)	Fully human IgG4 mAb
PD‐L1	Atezolizumab (Tecentriq®)	Humanized IgG1 mAb
	Avelumab (Bavencio®)	Fully human IgG1 mAb
	Durvalumab (Imfinzi®)	Fully human IgG1 mAb
AXL (TAM RTK)	Cabozantinib (Cabometyx®)	Small molecule inhibitor (also inhibits c‐MET, VEGFR2, and MET)
	Gilteritinib (Xospata®)	Small molecule inhibitor (also inhibits FLT3)
CD38	Daratumumab (Darzalex®)	Fully human IgG1 mAb
	Isatuxumab (Sarclisa®)	Chimeric murine/human IgG1 mAb

Despite the success of ICB therapy to date, there remains a significant population of patients who receive no clinical benefit from treatment with immune checkpoint inhibitors. One major factor that has been associated with response to checkpoint blockade is the presence of a preexisting immune response to the tumor, such that there is a population of T lymphocytes amenable to the effects of checkpoint inhibition.^335^, ^336^, ^337^, ^338^ This prerequisite means that patients who do not mount natural immune responses to their tumors are inherently poor candidates for ICB therapy. Pre‐immunization of such immunologically naïve individuals with DC‐based vaccines that induce T cell responses to tumor Ag has therefore become an appealing strategy for increasing the percentage of patients likely to benefit from ICB regimens. Though exogenous DC vaccines and DNA/peptide/protein‐based vaccines that rely on Ag processing and presentation by endogenous DC have had minimal efficacy as monotherapies in cancer patients to date,^75^ these approaches have also likely been reciprocally limited by checkpoint inhibition of the T cell responses they induce. Therefore, combining DC‐based and ICB therapies may indeed yield synergistic antitumor and clinical benefits, as vaccination could induce an antitumor T cell response capable of being supported by checkpoint blockade. To this point, a recent study found that autologous DC vaccination of metastatic melanoma patients yielded a T cell‐inflamed TME from tumors that had previously been characterized as immunologically “cold.”^339^ Of note, concurrent with the vaccine‐induced transition from “cold” to “hot” tumors, PD‐L1 expression was increased on tumor cells, and intratumoral CD8^+^ T cells were found not to express granzyme B. These findings support the aforementioned notion that DC‐based therapy alone may be compromised by tumor‐associated suppression of vaccine‐induced T cells but that checkpoint blockade might prevent tumor escape from such a vaccine‐induced response. As proof of principle, studies in murine models have shown that combining DC vaccination with checkpoint blockade therapy enhances TIL effector function and control of tumors.^340^, ^341^ A recent report from a Phase I clinical trial of gastric cancer patients also documented a reversal of dysfunction in vaccine‐induced CD8^+^ T cells when these cells were re‐stimulated in the presence of blocking antibodies against various checkpoint receptors, suggesting that a combination of DC vaccination and checkpoint blockade could also have therapeutic benefit in patients.^342^ Similar results have been obtained with therapeutic strategies that rely on the activity of endogenous DC. Peptide vaccination of advanced melanoma patients elicits CD8^+^ T cell responses that are restrained by PD‐1 and TIM‐3, and blockade of these receptors during ex vivo restimulation enhances their proliferative capacity and effector cytokine production.^343^ Likewise, regimens that promote cDC1 expansion and activation in tumors^9^, ^344^, ^345^, ^346^ or that target tumor Ag to this particular DC subset^347^ are also supported by checkpoint blockade.

As described herein, a handful of checkpoint pathways typically associated with the regulation of T cell immunity are also shared by DC. It is therefore likely that currently approved checkpoint inhibitors targeting these particular pathways improve antitumor T cell responses not only via direct influences on T cells but also by indirect mechanisms that involve enhanced DC function, as has recently been shown in a murine tumor model.^348^ In addition to the benefits of targeting these pathways, the identification of other innate‐specific immunologic checkpoints now offers a new array of targets for inhibitors that specifically engage innate populations such as DC. Targeting these innate checkpoints in conjunction with what have classically been viewed as T lymphocyte checkpoints is emerging as an attractive way of supporting both the induction and maintenance of antitumor T cell responses. For instance, several preclinical studies have demonstrated the therapeutic efficacy of regimens that concomitantly target CD47 and the PD‐1/PD‐L1 or CTLA‐4 axes.^349^, ^350^, ^351^, ^352^, ^353^, ^354^ As anticipated, mechanistic insights from many of these studies have shown that the enhanced phagocytosis and immune sensing of tumor cells exhibited by DC following CD47 blockade promotes tumor Ag cross‐presentation and the activation of antitumor T cells whose effector functions are then bolstered by interference with the CTLA‐4 and PD‐1 pathways. With the multitude of phagocytic and innate immune sensing checkpoints discovered to date (Figure 1), targeting other combinations of innate and adaptive checkpoints pathways may also broaden the reach and efficacy of ICB therapy for cancer patients.

Though combination therapies targeting both DC and T lymphocytes can yield more robust immune responses than monotherapies, metabolic suppression within the TME remains a significant barrier to the antitumor immune function of these cells and can limit the efficacy of immune responses achieved by vaccination, ICB, adoptive transfer regimens, or any combination thereof. Therefore, therapeutic strategies that also consider ways of improving the metabolic fitness of DC and T lymphocytes are also emerging as effective approaches to support cancer immunotherapies. While many of the checkpoint pathways described in this review regulate immunometabolism themselves, it is also possible to combine ICB or other therapies with more direct approaches to improve metabolic conditions for tumor‐associated DC and T cells. As an example, accumulation of serum kynurenine as a result of the IDO/TDO‐tryptophan‐kynurenine pathway is a poor prognostic factor for the overall survival of melanoma and renal cell carcinoma patients treated with nivolumab,^355^ suggesting that interference with this pathway might improve patient response to PD‐1 and possibly other checkpoint inhibitors. To this point, inhibitors of both IDO and TDO have both recently been shown to improve the antitumor efficacy of ICB therapies in murine models,^356^, ^357^, ^358^ as has pharmacologic inhibition of either signaling or metabolic pathways that promote IDO expression in DC.^98^, ^359^ Likewise, multiple approaches to augment immunotherapy by interfering with adenosine‐mediated immune suppression have also been explored. In murine models, antibody blockade and pharmacological inhibition of either ectonucleotidases that generate adenosine from extracellular ATP or A_2A_ receptors that transmit suppressive signals from adenosine have both been shown to enhance the antitumor efficacy of checkpoint blockade and ACT therapies.^328^, ^330^ These strategies are currently being investigated in clinical settings as well, and their potential is highlighted by results from a recent Phase I trial demonstrating immunological and clinical benefits of combination therapy with the A_2A_R antagonist ciforadenant and the PD‐L1 inhibitor atezolizumab in treatment‐refractory renal cell carcinoma patients.^324^ Similar combinatorial approaches that aim to offset the immunosuppressive effects of other oncometabolites such as lactate or that drive immune‐promoting metabolic pathways in DC and T cells are also being investigated in preclinical and clinical settings. Examples of these and other types of combination therapies designed to support the immunologic functions of DC and/or T lymphocytes and that have reached clinical stages of development are highlighted in Table 2. Though by no means a comprehensive list, the select trials described here provide a snapshot of the types of combinatorial approaches made possible by recent advances in our understanding of immune regulation within the TME, and it will be exciting to follow the outcomes of these and related trials in the months and years ahead.

**TABLE 2:** Select clinical trials investigating combinatorial therapies that target/interfere with immunologic/metabolic checkpoints

Innate + Adaptive Immune Checkpoint Blockade					
Treatment	Cancer(s)	Trial	Phase	Status	Rationale
TTI‐621 + α‐PD‐1/PD‐L1	Various solid tumors	NCT02890368	I	Recruiting	TTI‐621 is a SIRPα‐IgG1 recombinant fusion protein that binds CD47. May enhance DC‐mediated T cell responses that can be supported by PD‐1/PD‐L1 blockade.
ALX148 + Pembrolizumab	Advanced solid tumors, Non‐Hodgkin lymphoma	NCT03013218	I	Recruiting	ALX148 is a SIRPα‐IgG1 recombinant fusion protein that binds CD47. May enhance DC‐mediated T cell responses that can be supported by PD‐1 blockade.
BI 765063 + BI 754091	Advanced solid tumors	NCT03990233	I	Recruiting	BI 765063 is an α‐SIRPα mAb, and BI 754091 is an α‐PD‐1 mAb. May enhance DC‐mediated T cell responses that can be supported by PD‐1 blockade.

Immunologic + Metabolic Checkpoint Blockade					
Treatment	Cancer(s)	Trial	Phase	Status	Rationale
Ciforadenant + Atezolizumab	RCC, Metastatic castration‐resistant prostate cancer	NCT02655822	I	Recruiting	Ciforadenant is a small molecule antagonist of A_2A_R that may prevent adenosine‐mediated suppression of T cells supported by PD‐L1 blockade.
Daratumumab + Nivolumab	Pancreatic cancer, NSCLC, TNB	NCT03098550	I/II	Active, not recruiting	Daratumumab is an α‐CD38 mAb that blocks conversion of ATP to adenosine. May enhance immunogenic ATP signaling in DC and prevent adenosine‐mediated suppression of T cells supported by PD‐1 blockade.
TTX‐030 + Pembrolizumab	Solid tumors, Lymphoma	NCT03884556	I	Recruiting	TTX‐030 is an α‐CD39 mAb that blocks conversion of ATP to adenosine. May enhance immunogenic ATP signaling in DC and prevent adenosine‐mediated suppression of T cells supported by PD‐1 blockade.
Oleclumab + Durvalumab + Chemotherapy	TNBC	NCT03616886	I/II	Recruiting	Oleclumab is an α‐CD73 mAb that may prevent adenosine‐mediated suppression of T cells supported by PD‐L1 blockade.
INCB001158 + Pembrolizumab	Advanced/metastatic solid tumors	NCT02903914	I/II	Recruiting	INCB001158 is an arginase inhibitor that may prevent arginine metabolism‐associated suppression of T cells supported by PD‐1 blockade.
BMS‐986205 + Nivolumab + Radiation ± Chemotherapy	Glioblastoma	NCT04047706	I	Recruiting	BMS‐986205 is an IDO1 inhibitor that may prevent tryptophan metabolism‐associated suppression of T cells supported by PD‐1 blockade.
Indoximod + Ipilimumab, Nivolumab, or Pembrolizumab	Unresectable stage III/IV melanoma	NCT02073123	I/II	Active, not recruiting	Indoximod is a tryptophan mimetic that inhibits IDO/TDO activity and may therefore prevent tryptophan metabolism‐associated suppression of T cells supported by CTLA‐4 or PD‐1 blockade.
BMS986205 + Nivolumab + Relatlimab	Advanced or metastatic solid cancers	NCT03459222	I/II	Recruiting	BMS986205 is an IDO1 inhibitor that may prevent tryptophan metabolism‐associated suppression of T cells supported by combination PD‐1/LAG‐3 blockade.

Vaccination + Immunologic/Metabolic Checkpoint Blockade					
Treatment	Cancer(s)	Trial	Phase	Status	Rationale
Peptide‐pulsed autologous DC + Pembrolizumab + Cycolphosphamide	Stage III/IV melanoma	NCT03092453	I	Recruiting	Cyclophosphamide to deplete Tregs, followed by DC vaccine to induce a melanoma Ag‐specific T cell response that can be supported by PD‐1 blockade.
Tumor lysate‐pulsed autologous DC + Poly ICLC + Pembrolizumab	Recurrent glioblastoma	NCT04201873	I	Recruiting	Poly ICLC may enhance the immunogenicity of DC vaccine, inducing a tumor Ag‐specific T cell response that can be supported by PD‐1 blockade.
mRNA vaccine (BI 1361849) + Durvalumab ± Tremelimumab	NSCLC	NCT03164772	I/II	Recruiting	BI 1361489 is a self‐adjuvanted mRNA vaccine that may elicit T cell responses capable of being supported by PD‐L1 blockade (durvalumab) or a combination of PD‐L1 and CTLA‐4 blockade (tremelimumab).
TLPLDC Vaccine + Standard‐of‐care ICB	Stage IV melanoma	NCT02678741	I/II	Active, not recruiting	TLPLDC (tumor lysate, yeast particle‐loaded DC) vaccine may induce T cell responses whose antitumor efficacy may be enhanced by an approved immune checkpoint inhibitor.
Tergenpumatucel‐L vaccine + Docetaxel + Indoximod	NSCLC	NCT02460367	I	Active, not recruiting	Tergenpumatucel‐L is a vaccine comprised of allogeneic NSCLC cells genetically modified to express a carbohydrate to which humans have pre‐existing immunity. The tryptophan mimetic indoximod may improve the efficacy of standard vaccination and chemotherapy by preventing IDO/TDO‐mediated immune suppression.

Adoptive Cell Transfer Therapy + Immune Checkpoint Blockade/Interference					
Treatment	Cancer(s)	Trial	Phase	Status	Rationale
Autologous TIL/IL‐2 + Nivolumab	Stage IIIb/IIIc/IV melanoma	NCT03374839	I/II	Recruiting	α‐PD‐1 ICB therapy may enhance the antitumor efficacy of adoptively transferred TIL by preventing PD‐1‐mediated immune suppression.
Autologous TIL/IL‐2 + Nivolumab + Lymphodepleting chemotherapy	NSCLC, SCC, Adenosquamous carcinoma, Adenocarcinomas	NCT03215810	I	Active, not recruiting	α‐PD‐1 ICB therapy may enhance the antitumor efficacy of adoptively transferred TIL by preventing PD‐1‐mediated immune suppression.
Autologous TIL/IL‐2 + Ipilimumab + Nivolumab	Metastatic melanoma	NCT03526185	I	Recruiting	The antitumor efficacy of adoptively transferred TIL may be improved by combinatorial blockade of the CTLA‐4 and PD‐1 checkpoints.
NYCE T cells with CRISPR‐edited TCR and PD‐1 + Lymphodepleting chemotherapy	Multiple myeloma, Melanoma, Synovial sarcoma, Myxoid/round cell liposarcoma	NCT03399448	I	Active, not recruiting	NYCE T cells are autologous NY‐ESO‐1 redirected T cells that have been CRISPR‐edited to eliminate expression of endogenous TCR and PD‐1. May improve efficacy of adoptively transferred tumor Ag‐specific T cells by preventing PD‐1‐mediated suppression.
EGFRvIII‐redirected CAR‐T cells + Pembrolizumab	EGFRvIII+, MGMT‐unmethylated glioblastoma	NCT03726515	I	Active, not recruiting	PD‐1 blockade may enhance the antitumor activity of autologous CAR‐T cells engineered to recognize EGFRvIII+, MGMT‐unmethylated glioblastomas that are poorly responsive to standard‐of‐care alkylating chemotherapy.
MUC1‐redirected CAR‐T cells with CRISPR‐mediated PD‐1 KO	Advanced NSCLC	NCT03525782	I	Recruiting	Interference with PD‐1‐mediated immune suppression by CRISPR gene editing may improve the antitumor efficacy of adoptively transferred MUC1‐redirected CAR‐T cells.

Abbreviations: KO, knockout; NSCLC, non‐small cell lung cancer; RCC, renal cell carcinoma; SCC, squamous cell carcinoma; TNBC, triple negative breast cancer.

## 8 CONCLUSIONS

Over the past two decades, the field of oncology has been witness to an unparalleled transformation in our understanding of the immune system in the context of cancer. Insights into the basic immunobiology of DC and T lymphocytes have revealed an exquisite cooperation between these cells over the course of an antitumor immune response, and understanding how the activity of these cells is controlled by both immunologic and metabolic factors has shaped our appreciation for the diverse mechanisms regulating immune function within the TME. The complexity of this regulation cannot be overstated, with an array of immunologic and metabolic checkpoints now being recognized as potential barriers to antitumor immune function. At the same time, this complexity has also shed light on a multitude of potential targets for therapeutic interventions that aim to promote metabolic fitness and antitumor immune reactivity by both DC and T cells. The therapeutic success of many of these interventions in preclinical settings has paved the way for significant advances in the treatment of cancer patients, yielding never‐before‐seen rates, and duration, of clinical response. Moreover, insights into both innate and acquired mechanisms of resistance to these approaches have pointed towards a new age of combinatorial therapies that is already paying significant dividends in the clinical management of diverse cancer types. Though there will certainly be no one‐size‐fits‐all approach to these next‐generation combinatorial regimens, thorough biomarker analyses at the time of initial cancer diagnosis will in many cases reveal the ideal cohort of targets necessary to inform optimal therapeutic design. With this information in hand, the broad armamentarium of drugs and strategies either already available or currently in development will soon enable implementation of perhaps the most sophisticated forms of precision medicine ever seen in the field of immuno‐oncology, and it is expected that these approaches will continue to revolutionize cancer therapy in both the near and distant future.

## CONFLICT OF INTEREST

The author has declared no conflict of interest.
